# Insight into the Phylogenetic Relationships among Three Subfamilies within Heptageniidae (Insecta: Ephemeroptera) along with Low-Temperature Selection Pressure Analyses Using Mitogenomes

**DOI:** 10.3390/insects12070656

**Published:** 2021-07-19

**Authors:** Xiao-Dong Xu, Jia-Yin Guan, Zi-Yi Zhang, Yu-Rou Cao, Yin-Yin Cai, Kenneth B. Storey, Dan-Na Yu, Jia-Yong Zhang

**Affiliations:** 1 College of Chemistry and Life Science, Zhejiang Normal University, Jinhua 321004, China; KI01110015@163.com (X.-D.X.); a1345413239@163.com (J.-Y.G.); cixi55@126.com (Z.-Y.Z.); 13306760612@163.com (Y.-R.C.); caiyinyin122@163.com (Y.-Y.C.); 2 Department of Biology, Carleton University, Ottawa, ON K1S 5B6, Canada; kenneth.storey@carleton.ca; 3 Key Lab of Wildlife Biotechnology, Conservation and Utilization of Zhejiang Province, Zhejiang Normal University, Jinhua 321004, China

**Keywords:** Heptageniidae, mitochondrial genome, gene rearrangement, phylogenetic relationship, non-coding region (NCR), selective stress analysis

## Abstract

**Simple Summary:**

The Ephemeroptera is an ancient lineage of insects, among which the Heptageniidae is one of the most species-rich families, although its phylogenetic relationships have been controversial. The mitogenomes of Heptageniidae were found gene rearrangements of CR*-I-M-Q-M-ND2* and a conserved intergenic gap between *trnA* and *trnR.* Thus, 15 complete and two nearly complete mitogenomes of Heptageniidae were used to explore mitogenome structures and clarify the disputes of phylogenetic relationships among Heptageniidae. Additionally, the Heptageniidae samples collected from habitats with significant temperature differences were applied to investigate the adaptive evolution of mitochondrial PCGs under low-temperature stress.

**Abstract:**

We determined 15 complete and two nearly complete mitogenomes of Heptageniidae belonging to three subfamilies (Heptageniinae, Rhithrogeninae, and Ecdyonurinae) and six genera (*Afronurus*, *Epeorus*, *Leucrocuta*, *Maccaffertium*, *Stenacron*, and *Stenonema*). Species of Rhithrogeninae and Ecdyonurinae had the same gene rearrangement of CR*-I-M-Q-M-ND2*, whereas a novel gene rearrangement of CR-*I-M-Q*-NCR-*ND2* was found in Heptageniinae. Non-coding regions (NCRs) of 25–47 bp located between *trnA* and *trnR* were observed in all mayflies of Heptageniidae, which may be a synapomorphy for Heptageniidae. Both the BI and ML phylogenetic analyses supported the monophyly of Heptageniidae and its subfamilies (Heptageniinae, Rhithrogeninae, and Ecdyonurinae). The phylogenetic results combined with gene rearrangements and NCR locations confirmed the relationship of the subfamilies as (Heptageniinae + (Rhithrogeninae + Ecdyonurinae)). To assess the effects of low-temperature stress on Heptageniidae species from Ottawa, Canada, we found 27 positive selection sites in eight protein-coding genes (PCGs) using the branch-site model. The selection pressure analyses suggested that mitochondrial PCGs underwent positive selection to meet the energy requirements under low-temperature stress.

## 1. Introduction

As a primitive group of winged insects, Ephemeroptera comprises 40 families, 460 genera, and 3700 species [1,2,3]. Ephemeroptera is characterized by multiple ancestral signs including extra appendages (seven pairs of gills on larvae along with the forceps and long tails of adults), a unique prometamorphosis development pattern, and wings that do not fold flat over the abdomen, which have been intensely studied in phylogeny and historical processes [4,5]. As one of the most species-rich families among Ephemeroptera, Heptageniidae consists of three subfamilies (Ecdyonurinae, Heptageniinae, and Rhithrogeninae), 37 genera, and 606 described species [1,3,6]. Much effort has been made to figure out the taxonomy and phylogeny of Ephemeroptera using morphology features, molecular proofs, and combined data [7,8,9,10,11,12,13,14]. Nonetheless, the phylogenetic relationships within Heptageniidae remain controversial [6,13]. The phylogenetic systems of both McCafferty [9] and Kluge [10,11] supported Heptageniidae as belonging to Branchitergalia (Heptagenioidea) with a close relationship to Isonychiidae. The phylogenetic relationship developed using combined data of morphological characters and several nuclear genes by Ogden et al. [12] was different from the former hypotheses, supporting Heptageniidae as a monophyletic group, but its phylogenetic position remained uncertain. According to the newly published study by Ogden et al. [15], the phylogenetic results supported Heptageniidae as a sister group to Isonychiidae using over 440 targeted genomic protein coding regions (exons). In addition, the internal phylogenetic relationships within Heptageniidae were divided into three subfamilies (Heptageniinae, Rhithrogeninae, and Ecdyonurinae) and their relationship was shown as (Ecdyonurinae + (Heptageniinae + Rhithrogeninae)) by Wang and McCafferty [13] and Webb and McCafferty [6]. By contrast, the phylogenetic analysis was presented as (Rhithrogenidae + (Ecdyonurinae + Heptageniinae)) by Ogden et al. [15]. In addition, the genera *Stenonema* was redefined to include *Maccaffertium* via two mitochondrial genes (*COX1* and *16S rRNA*) and two nuclear genes (*Wingless* (*Wg*) and *histone H3*) by Zembrzuski and Anderson [16].

The typical mitochondrial genome (mitogenome) of insects is a 14–20 kb double-stranded circular piece of DNA [17,18,19]. It encodes 37 genes including 13 protein-coding genes (PCGs), two ribosomal RNAs (rRNAs, *16S rRNA* and *12S rRNA*), 22 transfer RNAs (tRNAs), and the A + T-rich region (control region, CR). Since the mitogenome has features such as rapid evolution rates, small genome sizes, and relatively low recombination and maternal inheritance, it is considered to be an excellent molecular marker for studies in phylogeny, evolution, and comparative genomics [18,20,21,22]. Although most insect mitogenomes are conservative, gene rearrangements and long non-coding regions (NCRs) have been variously reported in Coleoptera, Hemiptera, Lepidoptera, Mantodea, Orthoptera, Thysanoptera, etc. [19,23,24,25,26,27,28,29,30,31]. According to published reports, gene rearrangements of tRNA genes including duplication, translocation, and pseudogenization were mainly concentrated in the regions of CR*-I-Q-M-ND2*, *COX1-K-D-ATP8*, and *ND3-A-R-N-S-E-F-ND5* [31,32].

Within the order Ephemeroptera, most species retain the same 37 genes as the hypothesized ancestral mitogenome of insects except for Siphluriscidae, Baetidae, Leptophlebiidae, Ephemerellidae, and Heptageniidae [32,33,34,35,36,37,38,39,40,41]. The mitogenome of *Siphluriscus chinensis* (Siphluriscidae) encoded an extra *trnK* located between *trnS* and *trnE* in the minor coding strand [36]. The *trnC* and *trnY* in *Alainites yixiani* (Baetidae) translocated from a position between *trnW* and *COX1* into the gene cluster of *I-Q-M*, and the gene order was rearranged as *I-C-Q-Y-M*. Furthermore, one copy of inversion and translocation of *trnI* was detected in *Ephemerella* sp. Yunnan-2018, *Ephemerella* sp. MT-2014, *Serratella zapekinae*, *Serratella* sp. Liaoning-2019, and *Serratella* sp. Yunnan-2018, along with three duplicate copies of inversion and translocation of *trnI* in *Torleya grandipennis* and *T. tumiforceps* (Ephemerellidae) [32]. The *trnA* and *trnR* genes switched positions in *Habrophlebiodes zijinensis* (Leptophlebiidae) resulting in a gene arrangement *R-A-N-S-E-F*. Within the family Heptageniidae, an extra *trnM* was observed in the location between *trnQ* and *ND2*; thus, the gene arrangement was arranged as *I-M-Q-M* in *Epeorus herklotsi*, *Epeorus* sp. JZ-2014, *Epeorus* sp. MT-2014, and *Parafronurus youi* [33,34,35]. Surprisingly, no gene rearrangements were found in *Paegniodes cupulatus* (Heptageniidae), showing that its gene order was conservative and different from other Heptageniidae species.

Despite the fact that mitogenomes are generally considered to be under neutral or nearly neutral selection [42], several studies have pointed out that positive selection acted on mitochondrial PCGs linked to environmental adaptations [43,44,45]. In this way, as a potential target associated with energy metabolism under environmental selection pressure, the mitogenome may be suitable for studying positive selection or natural selection. According to the mitochondrial PCGs of flying and flightless grasshoppers, a significant positive selection was found in several genes including *ND2*, *ND4*, *ND4L*, *ND5*, *ND6*, *ATP8*, and *COX3* in flying lineages [45]. Hence, this indicated that positive selection stimulated mitochondrial genes to better suit the energy demands of flight in grasshoppers. Likewise, the mitochondrial PCGs were affected by positive selection from the last common ancestor of Pterygota and flying insects, which illustrated that those mitochondrial PCGs related to energy metabolism underwent adaptive evolution during the evolution of flight capacity in insects [45]. As aquatic insects, mayflies spend most of their developmental stages in the water. Among various environmental factors, the water temperature was shown to be crucial to the morphology, behavior, growth, and life cycle of mayflies [46,47]. Therefore, mayflies were proposed as appropriate models to investigate the adaptive evolution of aquatic insects in a low-temperature environment.

To explore the characteristics of gene rearrangements and the phylogenetic relationship of subfamilies in Heptageniidae, we determined the mitogenomes of 17 species from all three subfamilies and six genera of Heptageniidae. The phylogenetic relationship within Ephemeroptera was constructed with gene rearrangements and the location of NCRs to clarify the phylogenetic controversies. Moreover, samples of several Heptageniinae (*Maccaffertium*, *Stenacron*, and *Stenonema*) and *Leucrocuta* were collected from Ottawa, Canada. The climate there is so cold that the lowest temperature is below 0 °C and water temperature is below 10 °C for eight months of the year [48]. Thus, these mayfly nymphs had to be exposed to low water temperature for a long time. Other samples of *Epeorus* and *Afronurus* were from southern provinces (Zhejiang and Yunnan) of China where the mean annual water temperature was about 24–26 °C. Accordingly, Heptageniidae samples collected from habitats with significant temperature differences were suitable materials to assess the adaptive evolution of mitochondrial PCGs under low-temperature stress. In brief, our research not only provides novel insight into the gene rearrangements and phylogenetic relationships within Heptageniidae, but also investigates the evolutionary mechanisms of aquatic insect mitochondrial PCGs under low-temperature stress.

## 2. Materials and Methods

### 2.1. Sampling Collection and DNA Extraction

Six specimens were collected from the Rideau River, Ottawa, Canada in July 2017. Eleven specimens were collected from Wu River, Jinhua, Zhejiang province, and Chuan River, Jingdong, Yunnan province, China (Table 1). The specimens were all identified on the basis of a combination of nymph morphology and the alignment of *COX1* genes. Because some new species and genera were found in this study, we only identified those species at the genus or family level (Table 1). Samples were stored in 100% ethanol at −40 °C in J.Y.Z.’s lab, College of Life Science and Chemistry, Zhejiang Normal University, China. Our study included 17 specimens representing all three subfamilies, nine specimens from the subfamily Ecdyonurinae (*Afronurus* and *Leucrocuta*), five specimens from Heptageniinae (*Maccaffertium*, *Stenacron*, and *Stenonema*), and two specimens from Rhithrogeninae (*Epeorus*). Total DNA was extracted from legs or half of the whole individual of every species using Ezup Column Animal Genomic DNA Purification Kit (Sangon Biotech Company, Shanghai, China).

### 2.2. PCR Amplification and Sequencing

This study used both normal polymerase chain reaction (PCR) and long-and-accurate PCR (LA PCR) methods with Takara Taq or Takara LA Taq DNA polymerase (Takara, Dalian, China). Normal PCR (product length < 3000 bp) or LA PCR (product length > 3000 bp) reaction conditions were as in Gao et al. [49]. The mitogenomes were amplified in 700–2000 bp short fragments with universal primers according to the method of Simon et al. [50,51] and Zhang et al. [33]. Afterward, we designed specific primers (Table S1, Supplementary Materials) with Primer Premier 5.0 [52] according to the obtained sequences. All PCR products were obtained in both forward and reverse directions using the primer-walking method and AB13730XL by Sangon Biotech Company (Shanghai, China).

### 2.3. Mitogenome Annotation and Sequence Analyses

We inspected and assembled mitochondrial sequences using DNASTAR Package v.7.1 [53]. All tRNA genes and their secondary structures were identified by MITOS (http://mitos.bioinf.uni-leipzig.de/index.py, accessed on 11 January 2021) [54]. Two rRNA genes (*12S* and *16S rRNA*) and 13 PCGs were determined by alignments with homologous mtDNA sequences from several species in Heptageniidae using Clustal X [55,56]. The nucleotide composition, codon usage, and relative synonymous codon usage (RSCU) were calculated using Mega 7.0 [56]. The GC skews and AT skews were separately calculated using the following formulae: AT skew = (A − T)/(A + T), and GC skew = (G − C)/(G + C) [57]. Tandem repeats in CRs were detected via Tandem Repeat Finder V 4.09 [58]. The secondary structures of NCRs were found and mapped via RNAstructure Web Servers [59].

### 2.4. Phylogenetic Analyses

Forty-nine species from Ephemeroptera, including 14 families (Table 2), were used in phylogenetic analyses of Heptageniidae and Ephemeroptera [32,33,34,35,36,37,38,39,40,41]. The taxon of *Siphluriscus* (Siphluriscidae) was recovered as a sister clade to all other mayflies and, therefore, *S. chinensis* from the family Siphluriscidae was selected as the outgroup [12,36]. Thirteen PCGs of mayfly mitogenomes were used to construct BI and ML phylogenetic trees. The nucleotide sequences of the 13 PCGs were used for DNA alignment by MAFFT v. 7.475 [60], and the conserved regions were detected by Gblock 0.91b using default settings [61]. The program PartionFinder 1.1.1 was employed on the basis of Bayesian information criterion (BIC) to identify the best partitioning scheme and substitution model, and all 12 partitions were observed (Table S2, Supplementary Materials) [62]. ML analysis was run in RAxML 8.2.0 with a GTRGAMMAI model, and branch support for each node was evaluated with 1000 replicates [63]. BI analysis was performed in MrBayes version 3.2 using a GTR + I + G model [64]. Each of four chains ran for 10 million generations, and sampling every 1000 generations was used for phylogenetic relationship reconstruction [64]. The convergence was evaluated using Tracer version 1.5, and trees from the first 25% of the samples were removed as burn-in during BI analysis.

### 2.5. Positive Selection Analysis

The software EasyCodeML [65] was used to evaluate the selective pressure on the PCGs of Heptageniidae mitogenomes. Due to the significantly lower environment temperatures experienced by the Heptageniidae species from Ottawa, Canada, these were selected as the foreground branch to investigate the molecular evolution trends of mitochondrial PCGs under low-temperature stress. Both the branch model and the branch-site model were employed to explore whether positive selection occurred on specific branches and specific sites at the branch [66,67]. The branch models were performed under the one-ratio model (M0) presuming that *ω* was fixed over all of the tree or the two-ratio model presuming that *ω* in specific branches was different from the rest of the tree. Moreover, the branch-site models were combined with heterogeneous *ω* across sites and branches, which allows positive selection along specified branches (Model A) and can be compared against a null model (Model A_null_) that allows neutral evolution and negative selection. Likelihood ratio tests (LRTs) and Bayes empirical Bayes (BEB) were used to assess these models and evaluate the posterior probability of positive selection sites, respectively. Additionally, information on the structure and function of the positively selected genes was acquired using UniProt [68], and 3D structures of the corresponding proteins were built by SWISS-MODEL Workspace [69].

## 3. Results

### 3.1. General Features of the Mitogenomes

**Figure 1:**
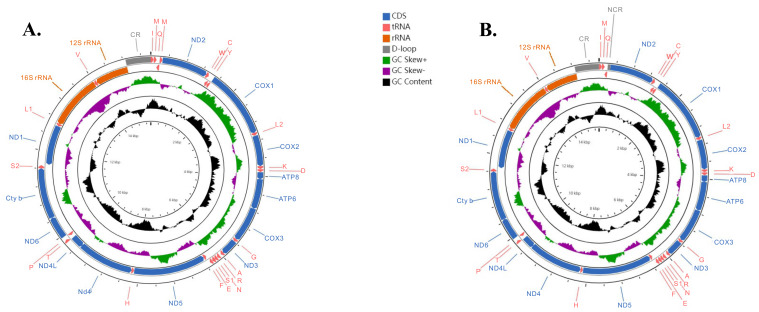
Circular visualization and organization of the complete mitogenome. External genes on the circle are encoded by the positive strand (5′→3′) and internal genes are encoded by the negative strand (3′→5′). (**A**) Mitogenomes in the subfamily Ecdyonurinae (*Afronurus* and *Leucrocuta*) and Rhithrogeninae (*Epeorus*); (**B**) mitogenomes in the subfamily Heptageniinae (*Maccaffertium*, *Stenacron*, and *Stenonema*).

The 17 mitogenomes of Heptageniidae used in this study ranged in length from 15,319 bp in *Maccaffertium mediopunctatum* (McDunnough, 1926) [70] to 15,883 bp in *Afronurus yixingensis* (Wu and You, 1986) [71] (Tables S3 and S4, Supplementary Materials) and encoded 13 PCGs, two rRNA genes, 22 or 23 tRNA genes (containing an extra *trnM* gene in some species), and one CR (Figure 1). Most of the genes were encoded on the major strand, also called the J strand, whereas the minor strand (N strand) carried the remaining genes (four PCGs and eight tRNAs). The A + T content, AT-skew, and GC-skew of corresponding regions (mitogenomes, PCGs, and rRNAs) were separately calculated for each mayfly species and shared conserved characteristics with others (Table 3). The tRNAs of these mayflies all showed the classical cloverleaf secondary structures.

All these mitochondrial PCGs used conventional invertebrate ATN as start codons, except for *COX1* which started with CCG in seven species: *Afronurus* (*A. furcata* (Zhou and Zhen, 2003) [72], *A. rubromaculata* (You, Wu, Gui, and Hsu, 1981) [73], *Afronurus* sp. LS53BF04, *Afronurus* sp. YW01BF06, *Afronurus* sp. 07BF85, *Afronurus* sp. 07BF86, and *Afronurus* sp. 07BF96). *ATP8* started with GTG in most mayflies except for *M. mediopunctatum* (McDunnough, 1926) [70], *M. modestum* (Banks, 1910) [13], *M. vicarium* (Walker, 1853) [74], *Stenacron interpunctatum* (Say, 1839) [75], *Stenonema femoratum* (Say 1823) [76], and *Afronurus* sp. YW01BF06. *ND2* started with GTG except for *Epeorus dayongensis* (Gui and Zhang, 1992) [77], *Epeorus* sp. LA03FY06, and *Afronurus* sp. 07BF96. *ND5* started with GTG except for *Leucrocuta aphrodite* (McDunnough, 1926) [70], and *ND6* started with GTT in *S. interpunctatum*. Typical stop codons TAA and TAG were observed in the majority of PCGs, whereas incomplete stop codons T or TA were assigned in *Cyt b* (*M. mediopunctatum*, *M. modestum*, *M. vicarium*, and *S. femoratum*), *COX2* (all species), *ND4* (all species), and *ND5* (all species). The codon number and RSCU in mitochondrial PCGs were conservative among these species (Table S5, Supplementary Materials).

The CRs of Heptageniidae mitogenomes ranged from 487 bp to 1037 bp, with the location between *12S rRNA* and *trnI*. Almost all CRs of these mitogenomes showed the highest A + T content compared to other regions (PCGs, rRNA genes, and tRNA genes) except for *A. furcata*, *A. yixingensis*, *Afronurus* sp. 07BF85, *Afronurus* sp. 07BF86, and *Afronurus* sp. LS53BF04. The A + T contents of these CRs ranged from 54.1% in *Afronurus* sp. 07BF86 to 77.8% in *Epeorus* sp. LA03FY06. The AT-skew values of the CRs were a little positive except for *A. yixingensis*, *Epeorus* sp. LA03FY06, and Heptageniidae sp. YW03BF02, whereas the GC-skew was strongly negative except for *Afronurus* sp. 07BF85, *Afronurus* sp. 07BF86, and *E. dayongensis*. Additionally, tandem repeats were detected in the CRs of *Afronurus* sp. 07BF85, *Afronurus* sp. 07BF86, *Afronurus* sp. LS53BF04, *A. furcata*, *A. rubromaculata*, *A. yixingensis*, and *Heptageniidae* sp. YW03BF02 (Figure S1, Supplementary Materials).

### 3.2. Gene Arrangements and NCRs

**Figure 2:**
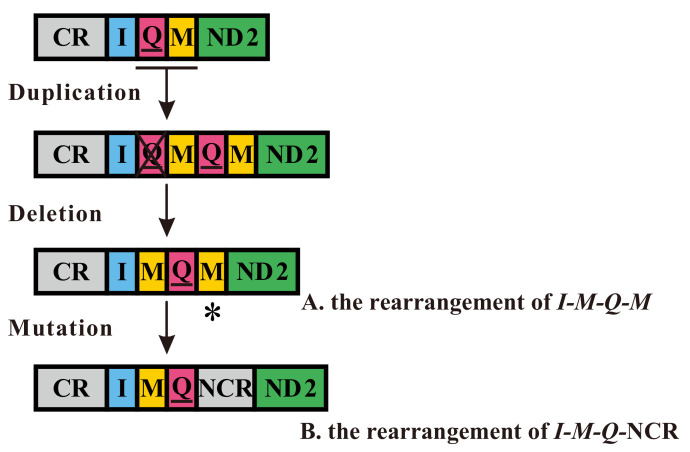
Proposed mechanism of gene rearrangements in the seventeen Heptageniidae mitogenomes. Gene sizes are not drawn to scale. Genes encoded by the L-strand are underlined, whereas those without underline are encoded on the H-strand. Different colored boxes represent different genes. The remaining genes and gene orders that were identical to the ancestral insect are left out. Horizontal lines, asterisk symbols, and crossed-out symbols represent gene duplications, gene mutations, and gene deletions, respectively. (**A**) Rearrangement of *I-M-Q-M*; (**B**) rearrangement of *I-M-Q-*NCR.

Two types of gene rearrangements occurred in the *I-Q-M* tRNA cluster and were found in all 17 freshly sequenced mitogenomes of Heptageniidae (Figure 2). The extra *trnM* was observed in the 11 mitogenomes of the subfamily Ecdyonurinae (*Afronurus* species and *L. aphrodite*) and Rhithrogeninae (*Epeorus* species). As for the location of two *trnM* copies, one was situated between *trnI* and *trnQ*, with another between *trnQ* and *ND2.* Thus, their tRNA cluster was shown as *I-M-Q-M*. The two copies of *trnM* genes showed high similarity (>70%) and had the same anti-codon (CAU) in nearly all species except for the second *trnM* (UAU) in *L. aphrodite* (Figure S2, Supplementary Materials). However, in mitogenomes of the subfamily Heptageniinae (*Maccaffertium* species, *S. interpunctatum*, and *S. femoratum*), a translocation of *trnM* was found and the *trnM* gene translocated into the position between *trnI* and *trnQ*. Furthermore, the NCR of 55–57 bp located between *trnQ* and *ND2* was detected in these species but showed low similarity to adjacent genes. Hence, the gene order in the species of Heptageniinae was shown as *I-M-Q-*NCR, and this is the first report of this novel gene rearrangement (*I-M-Q*-NCR) among mayfly mitogenomes.

The length, number, and distribution of the NCRs in these mitogenomes of Heptageniidae were relatively conservative. The number of NCRs in every mayfly species ranged from 7 to 12, whereas the length varied from 1 bp to 57 bp. Excluding the NCRs of short length (<15 bp) and the NCR located between *trnQ* and *ND2* (mentioned above), the NCRs located between *trnA* and *trnR* were observed in all Heptageniidae mitogenomes with lengths ranging from 25 bp (*M. vicarium*) to 47 bp (*E. herklotsi*). Interestingly, the NCRs could be folded as stem-loop secondary structures (Figure S3, Supplementary Materials) and were highly similar (>70%) to CR according to a comparison among the mitochondrial genomic sequences of most species (Figure S4, Supplementary Materials). Notwithstanding, the similarity between the NCR and CR was not exactly high (<70%) or the similarity sequence was short (<20 bp) in *A. furcata*, *A. rubromaculata*, *Afronurus* sp. 07BF86, *E. dayongensis*, *Epeorus* sp. LA03FY06, *M. mediopunctatum*, *M. modestum*, *M. vicarium*, *S. interpunctatum*, and *S. femoratum*. We also observed NCRs located between *trnS2* and *ND1* in all mitogenomes of Heptageniidae, which were of 16 bp in length.

### 3.3. Phylogenetic Analyses

**Figure 3:**
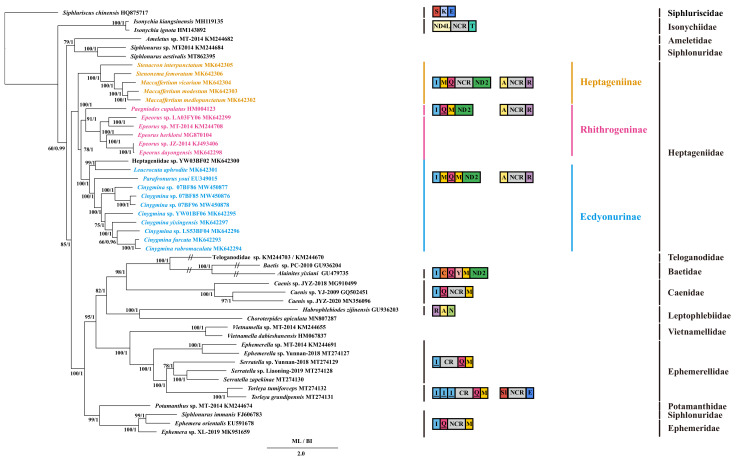
Phylogenetic tree of the relationships among 49 species of Ephemeroptera according to the nucleotide dataset of the 13 mitochondrial PCGs. *Siphluriscus chinensis* was used as the outgroup. The numbers above branches specify bootstrap percentages from ML (**left**) and posterior probabilities as determined from BI (**right**). The GenBank accession numbers of all species are shown in the figure. Box images on the right show gene rearrangements and the location of the NCR for the various mayfly species. Genes encoded by the minority strand are underlined, and those without underline are encoded by the majority strand. Different colored boxes represent different genes. The remaining genes and gene orders that were identical to the ancestral insect are left out. Gene sizes are not drawn to scale. The asterisks (*) by Siphlonuridae on the far-right side denote the separation of these sequences.

The BI and ML phylogenetic relationships showed identical topologies (Figure 3). However, long-branch attraction (LBA) has been observed in Baetidae (*Baetis* sp. PC-2010 and *Alainites yixiani*) and *Teloganodidae* sp. MT-2014; thus, their phylogenetic positions still remain uncertain. In general, the monophyly of most families was supported in these phylogenetic trees except for Ephemeridae and Siphlonuridae, but the availability of only one species in Ameletidae, Polymitarcyidae, and Teloganodidae restricted a discussion of their monophyly and phylogenetic relationships.

Within Ephemeroptera, Isonychiidae was a sister group to the other families, according to the phylogenetic topologies. Then, Ameletidae (*Ameletus* sp. MT-2014) and one branch of Siphlonuridae (*Siphlonurus aestivalis* and *Siphlonurus* sp. MT-2014) were found to be a sister group. Heptageniidae was supported as a sister clade to the remaining Ephemeroptera (Baetidae, Caenidae, Ephemerellidae, Ephemeridae, Leptophlebiidae, Potamanthidae, Teloganodidae, and Vietnamellidae). Potamanthidae was the sister clade to (Ephemeridae + *Siphlonurus immanis*), whereas the remaining families formed another large clade. (Ephemerellidae + Vietnamellidae) was supported as a sister clade to (Leptophlebiidae + (Caenidae + (Baetidae + Teloganodidae))).

Concentrating on the phylogenetic relationship within Heptageniidae, the monophyly of three subfamilies (Ecdyonurinae, Heptageniinae, and Rhithrogeninae) and the genera *Afronurus*, *Epeorus*, and *Maccaffertium* was supported. The branch of Heptageniidae was divided into three clades as follows: (Heptageniinae + (Rhithrogeninae + Ecdyonurinae)). The first branch of Heptageniinae supported (*S. interpunctatum* + (*S. femoratum* + *Maccaffertium* species)). Then, *Paegniodes cupulatus* and *Epeorus* species formed the second branch of Rhithrogeninae. The third branch of Ecdyonurinae supported ((Heptageniidae sp. YW03BF02 + *L. aphrodite*) + (*P. youi* + *Afronurus* species)). Significantly, the phylogenetic relationships coincided with the gene order and the location of the NCRs. The lineage of Ephemerellidae was consistent with rearrangements of the *trnI* gene. The NCRs located between *ND4L* and *trnT* were found in the branch of Isonychiidae along with the NCRs located between *trnQ* and *trnM* in the branch of Caenidae. Within Heptageniidae, these branches corresponded to different gene arrangements: *I-M-Q-*NCR in Heptageniinae and *I-M-Q-M* in the remaining species of Rhithrogeninae and Ecdyonurinae, except for *I-Q-M* in *P. cupulatus*.

### 3.4. Positive Selection Analysis

According to the branch model, 3722 amino-acid sites were used to analyze selective pressure on the basis of the alignment of 13 PCGs in 22 species of Heptageniidae. The results were as follows: *p* < 0.001, *ω*_0_ = 0.02021, *ω* = 0.02123 < 1, illustrating that the foreground branch (the Heptageniinae species from Ottawa, Canada) was not subject to positive selection (Table S6, Supplementary Materials). On the contrary, we observed that 27 amino-acid sites were under positive selection (*p* < 0.001, BEB value > 0.95) in the analyses of the branch-site models, of which five amino-acid sites were under highly positive selection (BEB value > 0.99) (Table 4). The 27 positive selection sites corresponding to the mitochondrial PCGs were divided into eight genes, namely, *COX1* (two sites), *Cyt b* (two sites), *ND1* (two sites), *ND2* (five sites), *ND3* (one site), *ND4* (two sites), *ND5* (six sites), and *ND6* (seven sites). Accordingly, the mitochondrial complex I was the main protein complex under selective pressure, including 23 positive selection sites. To determine the functional meaning of these positive selection sites, we explored the feature keys of eight positively selected PCGs from low-temperature branches. The majority of the positive selection sites located within or near to the functional domains of the proteins were encoded by these genes, 14 of which were situated in the protein transmembrane domain of the encoding genes, with an additional six positive selection sites situated in other domains of corresponding genes (Table 5, Figure S5, Supplementary Materials).

## 4. Discussion

### 4.1. Gene Arrangements and NCRs

The typical gene arrangement occurred in most mayfly mitogenomes, except for Siphluriscidae, Baetidae, Leptophlebiidae, Ephemerellidae, and Heptageniidae [32,33,34,35,36,37,38,39,40,41]. Gene rearrangements in these groups were mainly concentrated in the regions of CR-*I-Q-M-ND2* and *A-R-N-S-E-F*. In our study, the gene rearrangements in the mitogenomes of Heptageniidae were divided into two types: a gene arrangement of *I-M-Q-M* in the subfamily Ecdyonurinae (*Afronurus*, *Parafronurus*, and *Leucrocuta*) and Rhithrogeninae (*Epeorus*), and a novel gene arrangement of *I-M-Q*-NCR in the subfamily Heptageniinae (*Maccaffertium*, *Stenacron*, and *Stenonema*). Moreover, two copies of *trnM* genes had the same anti-codon (CAU) in almost all species excluding the second *trnM* (UAU) in *L. aphrodite*. The codon AUA is translated as Met in the invertebrate mitochondrial genetic code, like the normal codon AUG, as reported in the fruit fly *Drosophila melanogaster* [78,79]. Therefore, the second *trnM* with anti-codon (UAU) was considered to be functional in *L. aphrodite*. Furthermore, similar gene rearrangements occurring in the region of CR-*I-Q-M-ND2* were also reported in other orders of insects, e.g., *M-I-Q* tRNA cluster in Lepidoptera (*Manduca sexta*) [80], *Q-I-M* in Hemiptera (*Neuroctenus parus*) [81], and *I-I-I-I-I-Q-M* in Mantodea (*Schizocephala bicornis*) [31]. Consequently, the region of CR-*I-Q-M-ND2* is regarded as a hotspot for gene rearrangements in insects.

The tandem duplication–random loss (TDRL) model [82,83] was proposed and used to explain similar gene rearrangements in other insects [81,84]. Therefore, the TDRL model can be reasonably used to explain the gene rearrangements of Heptageniidae (Figure 2). The region of CR*-I-Q-M-ND2* was presumed to be the original gene arrangement. The mechanisms of gene rearrangement of *I-M-Q-M* were assumed to be as follows: a tandem duplication of *Q-M* happened, followed by random loss of the first *trnQ*, resulting in the gene order *I-M-Q-M*, as reported in Zhang et al. [33]. As for the gene rearrangement of *I-M-Q*-NCR, we propose that the tandem duplication of *Q-M* happened, followed by the random loss of the first *trnQ* and the mutation of the second *trnM*. Consequently, the translocation of *trnM* and the NCR located between *trnQ* and *ND2* was observed in the subfamily Heptageniinae (*Maccaffertium*, *Stenacron*, and *Stenonema*). Notwithstanding, the mitogenome of *P. cupulatus* showed the typical insect gene order and was different from other mitogenomes of Heptageniidae. The conservative gene order of *P. cupulatus* was proposed to occur as follows: the extra *trnM* between *trnI* and *trnQ* was lost from the ancestral *I-M-Q-M* type and, thus, formed the *I-Q-M* gene arrangement. Therefore, the gene arrangements among genera of Heptageniidae need further study.

Generally, insect mitogenomes are of high compaction with rare and short NCRs except for the CR [17]. The great majority of mayfly mitogenomes featured short NCR lengths. However, NCRs of 25–47 bp were located between *trnA* and *trnR* and observed in all mitogenomes of Heptageniidae [33,34,35]. This feature is rarely observed in other mayfly mitogenomes. Thus, this NCR located between *trnA* and *trnR* may be a synapomorphy for Heptageniidae. The NCRs can form stem-loop secondary structures (Figure S3, Supplementary Materials), which may contribute to the progress of replication slippage and then an increase in duplicate copies [85]. Furthermore, the NCR was proposed as an alternative replication origin for mtDNA [86,87]. As for the occurrence of the NCR, it was inferred to derive mainly from the corresponding CR because of the high similarity between the two (>70%), such as the complete sequence (37 bp) in *L. aphrodite* (similarity 94.59%) and the partial sequence (23 bp) in *P. youi* (similarity 100%) (Figure S4, Supplementary Materials). Considering the long distance between the NCR and CR, the recombination model may be more suitable to explain NCR production [88,89]. The creation of the NCR was presumed to occur as follows: a fragment containing the CR was cleaved out and then inserted into a location between *trnA* and *trnR*. Although there is a low similarity between the NCR and CR (<70%) or a short similar sequence (<20 bp) in several species (as mentioned in the results), the NCR was proposed to have evolved independently under relaxed selective pressure instead of evolving in concert with the CR [89]. In addition, the short NCR located between *trnS2* and *ND1* was detected in all Heptageniidae mitogenomes, which has also been reported in Ephemeroptera and other insects [23,32,33,34,49]. According to the alignments of these NCRs of all Heptageniidae species (Figure S6), a highly conserved motif of 16 bp (TACTTAAAAARKTCAR) may be the binding site of the transcription termination factor (DmTTF) [90].

### 4.2. Phylogenetic Analyses

Higher-level phylogenetic relationships within Ephemeroptera have not been generally accepted [8,9,10,11,12]. In our results, the BI and ML phylogenetic analyses shared congruent topologies (Figure 3). *S. chinensis*, the only species of Siphluriscidae, was deemed as the basal group of Ephemeroptera from the study of Ogden et al. [12] and Zhang et al. [33]. The next were Isonychiidae, Ameletidae, and one species of Siphlonuridae, as indicated by our results. The phylogenetic position of Isonychiidae was convincingly supported by Ogden et al. [12] as the primitive clade except for Siphluriscidae and Baetidae from topologies. Then, Heptageniidae was supported as a sister clade to the remaining Ephemeroptera by our results, contrary to the topologies constructed by Kluge [10,11] and McCafferty [8,9], as well as Ogden et al. [15], which suggested that Heptageniidae was sister to Isonychidae based on morphological characteristics and nuclear data, respectively. According to a comparison of our results and other studies, the phylogenetic position of Heptageniidae is still challenging to determine.

As for Heptageniidae, the monophyly of three subfamilies Ecdyonurinae, Heptageniinae, and Rhithrogeninae was supported, consistent with the research of Wang and McCafferty [13] and Webb and McCafferty [6]. Nevertheless, the internal phylogenetic classification within Heptageniidae in our study differed from Wang and McCafferty [13]. In our study, the phylogenetic relationship within Heptageniidae was shown as (Heptageniinae + (Ecdyonurinae + Rhithrogeninae)), opposite to the (Ecdyonurinae + (Heptageniinae + Rhithrogeninae)) presented in Wang and McCafferty [13] and (Rhithrogenidae + (Ecdyonurinae + Heptageniinae)) in Ogden et al. [15]. When the phylogenetic classification was combined with gene rearrangements and NCRs, there was a specific correlation. The gene rearrangement of *I-M-Q*-NCR was concentrated in Heptageniinae, and the gene arrangement of *I-M-Q-M* was observed in the remaining species of Rhithrogeninae and Ecdyonurinae except for *I-Q-M* in *P. cupulatus*. These results illustrated that Ecdyonurinae and Rhithrogeninae were closely related, and the two formed a sister group to Heptageniinae. Phylogenetic relationships highly congruent with gene rearrangements and NCR locations were also reported in other insects [91,92], which suggests that synapomorphic gene rearrangements and NCRs have formed continuously during evolution and could provide effective phylogenetic information. However, the phylogenetic relationship of the three subfamilies within Heptageniidae is also controversial due to the lack of other evidence in our study. In addition, concerning the taxonomy of *Stenonema*, *Stenacron* and *Maccaffertium*, compared to the research of Zembrzuski and Anderson [16], it is a pity that we could not draw a valid conclusion on the basis of our results because of the lack of sequences for these genera. Further morphological and molecular data are required to demonstrate a more exact phylogenetic relationship among Ephemeroptera.

In fact, the gene arrangement of *I-Q-M* was found only in *P. cupulatus* of Heptageniidae. This was confusing as to whether such a gene arrangement was specific to the genus *Paegniodes* or formed during the random mutation progress. More mayfly mitogenomes are expected to be sequenced, which will help to explore the types of gene arrangements and clear phylogenetic classifications within Heptageniidae.

### 4.3. Positive Selection Analyses

Adaptive evolution of mitochondrial genes under environmental pressure is supported by several studies [43,44,45]. Environmental temperature significantly influences energy requirements and metabolic adaptation, which is essential to mayflies as aquatic insects [46,47]. Multiple subunits of the mitochondrial complexes associated with oxidative phosphorylation are encoded by mitochondrial genes, with the exception of complex II [93]. In this way, positive selection of mitochondrial genes was proposed to be related to temperature and adaptation to the energy demands of mayflies.

Analysis of the branch model showed that there was no positive selection on the foreground branch. It was proposed that information indicating positive selection was possibly covered by continuous neutral evolution or negative selection at most sites in the gene sequence [94]. According to the branch-site model, 27 positive selection sites were found. It is worth noting that 23 of the positive selection sites were concentrated on the coding sequence of mitochondrial complex I. As the first large protein complex of the respiratory chain, complex I provides the proton power for ATP synthesis during electron transfer from NADH to ubiquinone via the transmembrane proton pump [95,96,97]. Therefore, complex I is essential for the energy metabolism of cells and drives more than one-third of the total energy production in the mitochondrion [87]. *ND1*–*ND6* subunits are regarded as the minimal assembly of complex I and form the core of the transmembrane region [98], with the *ND2*, *ND4*, and *ND5* genes proposed to be main candidates to harbor the proton pump [99]. The importance of complex I and its subunits can explain the reason for more positive selection sites in complex I than in other complexes. In addition, several positive selection sites were also observed in the subunits (*Cyt b* and *COX1*) of complex III and complex IV. *Cyt b* is the main transmembrane subunit of complex III and exerts a crucial function in ATP production [100]. Furthermore, complex IV has regulatory effects in the electron transport chain, and its subunit *COX1* starts the assembly process of complex IV [101]. Moreover, the positive selection sites in the eight PCGs were located in or close to the functional domains according to the structural analysis (Table 5). Consequently, the adaptive changes in amino acids at these positive selection sites, especially in the functional regions, were proposed to affect protein stability or even function [102,103]. On the whole, mitochondrial PCGs are related to energy metabolism and can experience positive selection to cope with energy needs under low-temperature stress.

## 5. Conclusions

Fifteen complete mitogenomes and two nearly complete mitogenomes of Heptageniidae were successfully determined. Gene rearrangements in these mitogenomes of Heptageniidae were divided into two types: one with the commonly reported gene order of *I-M-Q-M* in the subfamily Ecdyonurinae (*Afronurus*, *Parafronurus*, and *Leucrocuta*) and Rhithrogeninae (*Epeorus*), and the other with a novel gene order of *I-M-Q*-NCR in Heptageniinae (*Maccaffertium*, *Stenacron*, and *Stenonema*). These gene rearrangements were explained by the tandem duplication–random loss (TDRL) model. In addition, the NCRs located between *trnA* and *trnR* were found in all Heptageniidae species and inferred to be a synapomorphy for Heptageniidae. The phylogenetic relationships within Ephemeroptera were highly congruent with the gene rearrangements and the location of NCRs, supporting the monophyly of Heptageniidae and its internal phylogenetic relationship (Heptageniinae + (Ecdyonurinae + Rhithrogeninae)). The selection pressure analyses indicated that mitochondrial PCGs of mayflies underwent positive selection to cope with potential energy requirements under low-temperature stress.

## Figures and Tables

**Table 1:** Information on specimen sources of the samples used in this study and NCBI Genbank accession numbers.

Subfamily	Species	Specimen No.	Sampling Localities	Accession Number
Heptageniinae	*Maccaffertium mediopunctatum* (McDunnough, 1926)	03FY33	Ottawa, Canada	MK642302
	*Maccaffertium modestum* (Banks, 1910)	03FY62	Ottawa, Canada	MK642303
	*Maccaffertium vicarium* (Walker, 1853)	03FY39	Ottawa, Canada	MK642304
	*Stenacroninterpunctatum* (Say, 1839)	03FY34	Ottawa, Canada	MK642305
	*Stenonema femoratum* (Say 1823)	03FY36	Ottawa, Canada	MK642306
Ecdyonurinae	*Leucrocuta Aphrodite* (McDunnough, 1926)	03FY51	Ottawa, Canada	MK642301
	*Afronurus furcata* (Zhou and Zhen, 2003)	08BF03	Zhejiang, China	MK642293
	*Afronurus rubromaculata* (You, Wu, Gui, and Hsu, 1981)	08BF02	Zhejiang, China	MK642294
	*Afronurus* sp1. YW01BF06	01BF06	Zhejiang, China	MK642295
	*Afronurus* sp2. LS53BF04	53BF04	Zhejiang, China	MK642296
	*Afronurus yixingensis* (Wu and You, 1986)	06BF03	Zhejiang, China	MK642297
	*Afronurus* sp. 07BF85	07BF85	Yunnan, China	MW450876
	*Afronurus* sp. 07BF86	07BF86	Yunnan, China	MW450877
	*Afronurus* sp. 07BF96	07BF96	Yunnan, China	MW450878
Rhithrogeninae	*Epeorus dayongensis* (Gui and Zhang, 1992)	18BF01	Zhejiang, China	MK642298
	*Epeorus* sp. LA03FY06	03FY06	Zhejiang, China	MK642299
/	Heptageniidae sp. YW03BF02	03BF02	Zhejiang, China	MK642300

**Table 2:** List of Ephemeroptera mitogenomes used to construct phylogenetic trees.

Family	Genus	Species	Length (bp)	GenBank No.	References
Ameletidae	*Ameletus*	*Ameletus* sp. MT-2014	15,141	KM244682	[35]
Baetidae	*Baetis*	*Baetis* sp. PC-2010	14,883	GU936204	Unpublished
	*Takobia*	*Alainites yixiani*	14,589	GU479735	Unpublished
Caenidae	*Caenis*	*Caenis* sp. JYZ-2018	15,254	MG910499	[33]
		*Caenis* sp. JYZ-2020	15,392	MN356096	[37]
		*Caenis* sp. YJ-2009	15,351	GQ502451	Unpublished
Ephemerellidae	*Ephemerella*	*Ephemerella* sp. MT-2014	14,896	KM244691	[31]
		*Ephemerella* sp. Yunnan-2018	15,256	MT274127	[32]
	*Serratella*	*Serratella* sp. Liaoning-2019	15,523	MT274128	
		*Serratella* sp. Yunnan-2018	15,134	MT274129	
		*Serratella zapekinae*	15,703	MT274130	
	*Torleya*	*Torleya grandipennis*	15,523	MT274131	
		*Torleya tumiforceps*	15,330	MT274132	
Ephemeridae	*Ephemera*	*Ephemera orientalis*	16,463	EU591678	Unpublished
		*Ephemera* sp. XL-2019	15,314	MK951659	[35]
Heptageniidae	*Afronurus*	*Afronurus furcata*	15,420	MK642293	This study
		*Afronurus rubromaculata*	15,519	MK642294	This study
		*Afronurus* sp. 07BF85	15,473	MW450876	This study
		*Afronurus* sp. 07BF86	15,696	MW450877	This study
		*Afronurus* sp. 07BF96	15,491	MW450878	This study
		*Afronurus* sp. YW01BF06	15,360	MK642295	This study
		*Afronurus* sp. LS53BF04	15,866	MK642296	This study
		*Afronurus* *yixingensis*	15,883	MK642297	This study
	*Epeorus*	*Epeorus dayongensis*	15,337	MK642298	This study
		*Epeorus herklotsi*	15,502	MG870104	[30]
		*Epeorus* sp. JZ-2014	15,338	KJ493406	Unpublished
		*Epeorus* sp. MT-2014	15,456	KM244708	[31]
		*Epeorus* sp. LA03FY06	15,514	MK642299	This study
	*Leucrocuta*	*Leucrocuta aphrodite*	15,428	MK642301	This study
	*Maccaffertium*	*Maccaffertium mediopunctatum*	15,319	MK642302	This study
		*Maccaffertium modestum*	15,324	MK642303	This study
		*Maccaffertium vicarium*	15,324	MK642304	This study
	*Paegniodes*	*Paegniodes cupulatus*	15,715	HM004123	Unpublished
	*Parafronurus*	*Parafronurus youi*	15,481	EU349015	[29]
	*Stenacron*	*Stenacron interpunctatum*	15,330	MK642305	This study
	*Stenonema*	*Stenonema femoratum*	15,332	MK642306	This study
		Heptageniidae sp. YW03BF02	15,663	MK642300	This study
Isonychiidae	*Isonychia*	*Isonychia ignota*	15,105	HM143892	Unpublished
		*Isonychia kiangsinensis*	15,456	MH119135	[34]
Leptophlebiidae	*Choroterpides*	*Choroterpides apiculata*	15,199	MN807287	[36]
	*Habrophlebiodes*	*Habrophlebiodes zijinensis*	14,355	GU936203	Unpublished
Potamanthidae	*Potamanthus*	*Potamanthus* sp. MT-2014	14,937	KM244674	[35]
Siphlonuridae	*Siphlonurus*	*Siphlonurus aestivalis*	15,120	MT862395	Unpublished
		*Siphlonurus immanis*	15,529	FJ606783	Unpublished
		*Siphlonurus* sp. MT-2014	14,745	KM244684	[31]
Siphluriscidae	*Siphluriscus*	*Siphluriscus chinensis*	16,616	HQ875717	[32]
Teloganodidae	*/*	Teloganodidae sp.	12,435/	KM244703	[31]
			2817	KM244670	
Vietnamellidae	*Vietnamella*	*Vietnamella dabieshanensis*	15,761	HM067837	Unpublished
		*Vietnamella* sp. MT-2014	15,043	KM244655	[31]

**Table 3:** Base composition of 17 mayfly mitochondrial genomes, with a greater separation of each of the three Mitogenome-PGC-rRNA-CR groups and separate underlining to mark the three different groups.

Species Name	Mitogenome-PGC-rRNA-CR											
	A + T (%)				AT-Skew				GC-Skew			
*Afronurus furcata*	64.5	64.4	64.9	56.9	0.005	−0.198	−0.024	0.23	−0.215	−0.004	0.339	−0.16
*Afronurus rubromaculata*	64.5	64.6	65.0	65.9	0.009	−0.193	−0.025	0.23	−0.218	−0.010	0.327	−0.11
*Afronurus* sp. 07BF85	63.3	62.8	65.5	60.0	−0.03	−0.210	0.032	0.02	−0.16	−0.012	0.284	0.05
*Afronurus* sp. 07BF86	64.0	64.4	65.7	54.1	−0.006	−0.191	0.02	0.13	−0.202	−0.017	0.310	0.02
*Afronurus* sp. 07BF96	62.9	62.4	65.3	/	−0.027	−0.213	0.035	/	−0.16	−0.010	0.288	/
*Afronurus* sp. YW01BF06	64.6	64.7	64.4	/	0.012	−0.186	0.002	/	−0.225	−0.010	0.317	/
*Afronurus* sp. LS53BF04	63.3	63.3	64.7	60.1	0.012	−0.198	−0.022	0.16	−0.227	−0.010	0.329	−0.30
*Afronurus yixingensis*	65.0	65.1	66.0	63.2	0.003	−0.203	−0.002	−0.01	−0.218	−0.004	0.305	−0.21
*Epeorus dayongensis*	64.8	64.2	66.1	73.6	0	−0.191	0.012	0.04	−0.212	−0.024	0.269	0.01
*Epeorus* sp. LA03FY06	67.1	66.3	67.6	77.8	−0.014	−0.188	0.02	0.00	−0.24	−0.003	0.307	−0.19
*Leucrocuta aphrodite*	65	65.0	65.1	65.4	−0.001	−0.187	−0.001	0.02	−0.186	−0.001	0.284	−0.18
*Maccaffertium mediopunctatum*	61.7	61.5	60.7	65.9	−0.002	−0.190	0.015	0.02	−0.178	−0.045	0.234	−0.35
*Maccaffertium modestum*	61.3	61.1	60.7	64.0	0.004	−0.191	0.025	0.01	−0.177	−0.038	0.231	−0.33
*Maccaffertium vicarium*	62.3	62.3	61.8	65.4	0.005	−0.169	0.013	0.00	−0.172	−0.046	0.253	−0.25
*Stenacron interpunctatum*	59.7	59.5	58.8	61.7	0.021	−0.184	0.007	0.05	−0.19	−0.057	0.266	−0.24
*Stenonema femoratum*	62.1	61.9	61.1	66.4	0.005	−0.190	−0.011	0.01	−0.166	−0.039	0.234	−0.30
*Heptageniidae* sp. YW03BF02	64.2	64.1	64.3	68.5	−0.006	−0.191	−0.011	−0.09	−0.183	0.002	0.27	−0.19

**Table 4:** Positive selection analysis of mitochondrial protein-coding genes based on the branch-site model.

Tree	Model	Ln L	Estimates of Parameters					Model Compared	2ΔL	LRT *p*-Value	Positive Sites
ML	Model A	−121,931.169602	**Site class**	0	1	2a	2b	Model A vs. Model A null	17.1487	0.00004434	682 Q 0.988 *, 749 S 0.979 *, 1340 L 0.977 *, 1636 V 0.954 *, 1827 E 0.989 *, 1843 A 0.983 *, 2118 P 0.995 **, 2123 F 0.983 *, 2167 S 0.979 *, 2288 T 0.983 *, 2311 T 0.991 **, 2398 A 0.970 *, 2613 S 0.962 *, 2619 M 0.986 *, 3001 L 0.971 *, 3005 S 0.964 *, 3155 S 0.969 *, 3313 S 0.984 *, 3444 S 0.989 *, 3466 H 0.978 *, 3557 L 0.976 *, 3566 I 0.996 **, 3582 C 0.985 *, 3664 E 0.996 **, 3665 Q 0.984 *, 3679 I 0.993 **, 3712 Q 0.981 *
			Proportion	0.9141	0.0377	0.0463	0.0019				
			Background *ω*	0.0137	1.0000	0.0137	1.0000				
			Foreground *ω*	0.0137	1.0000	2.7648	2.7648				
	Model A Null	−121,939.505555	/								/

Note: * and ** indicate BEB values > 0.95 and > 0.99, respectively.

**Table 5:** The features and description of the positive selection sites detected in the mitochondrial PCGs of Heptageniidae species.

Genes	Positive Selection Sites	Amino Acids		BEB Value	Feature Key *	Description
		Foreground	Background			
*COX1*	408	N	Q/K	0.988 *	Domain	COX1
	475	A	S	0.979 *	Domain	COX1
*Cyt b*	62	T	L	0.977 *	Domain	CYTB_NTER
	358	T/I	V/T/I	0.954 *	Transmembrane	Helical
*ND1*	173	Y	E/K/Q	0.989 *	/	/
	189	S	A/T	0.983 *	Transmembrane	Helical
*ND2*	148	S/G	P/S	0.995 **	Transmembrane	Helical
	153	T/S	F	0.983 *	Transmembrane	Helical
	197	N	S/P/N	0.979 *	Domain	Proton_antipo_M
	318	G/N	S/L/T/A	0.983 *	/	/
	341	S	S/P/T/L	0.991 **	/	/
*ND3*	85	Q	A/N/S/T	0.970 *	/	/
*ND4*	183	S	G/S/K	0.962 *	Transmembrane	Helical
	189	G	M/L	0.986 *	Transmembrane	Helical
*ND5*	25	T	L	0.971 *	Transmembrane	Helical
	29	L/A	S	0.964 *	Transmembrane	Helical
	179	E/H	S/G/T	0.969 *	Transmembrane	Helical
	337	S	S/N/I/T	0.984 *	Domain	Proton_antipo_M
	468	L	S/V/I/A	0.989 *	Transmembrane	Helical
	490	F	N/H/Q/G/S	0.978 *	Domain	NADH5_C
*ND6*	3	T	L/F/M	0.976 *	/	/
	12	L	T/I	0.996 **	Transmembrane	Helical
	28	I/V	C/S/I/V	0.985 *	Transmembrane	Helical
	110	S	E/D	0.996 **	/	/
	111	D	Q	0.984 *	/	/
	125	P	N/T/I/G	0.993 **	Transmembrane	Helical
	158	N	Q/N	0.981 *	Transmembrane	Helical

Note: * and ** indicate BEB values > 0.95 and > 0.99, respectively.

## Data Availability

The data supporting the findings of this study are openly available in National Center for Biotechnology Information (https://www.ncbi.nlm.nih.gov), accession numbers were MK642293-MK642306 and MW450876-MW450878.

## Supplementary Materials

The following are available online at https://www.mdpi.com/article/10.3390/insects12070656/s1: Figure S1. Organization of the repeat regions in the CRs of Heptageniidae species; Figure S2. Alignment between two copies of *trnM* sequences in Heptageniidae species; Figure S3. The possible secondary structure of the NCRs located between *trnA* and *trnR*; Figure S4. Alignment between CRs and the NCRs located between *trnA* and *trnR* in Heptageniidae species; Figure S5. Distribution of mutations in the three-dimensional structure of *COX1*, *Cyt b*, *ND1*, *ND2*, *ND3*, *ND4*, *ND5*, and *ND6* genes for the Heptageniidae species from Ottawa, Canada; Figure S6. Alignment of the NCRs located between *trnS2* and *ND1* in Heptageniidae species; Table S1. Specific primers used to amplify the Heptageniidae mitogenomes; Table S2. The partition schemes and best-fit models selected; Table S3. Location of features in the mitogenomes of the subfamily Ecdyonurinae (*Afronurus*, *Parafronurus*, and *Leucrocuta*) and Rhithrogeninae (*Epeorus*); Table S4. Location of features in the mitogenomes of the subfamily Heptageniinae (*Maccaffertium*, *Stenacron*, and *Stenonema*); Table S5. The codon number and relative synonymous codon usage in mitochondrial PCGs; Table S6. Positive selection analysis of mitochondrial PCGs based on the branch model.
