# Supplementary material for: Insight into the Phylogenetic Relationships among Three Subfamilies within Heptageniidae (Insecta: Ephemeroptera) along with Low-Temperature Selection Pressure Analyses Using Mitogenomes
Source: Insects. 2021 Jul 19;12(7):656. doi: 10.3390/insects12070656 (PMC8307263; doi:10.3390/insects12070656)
Supplement: Supplementary file 1 [file insects-12-00656-s001.zip › Figure S1. tandem repeats in CR.pdf]

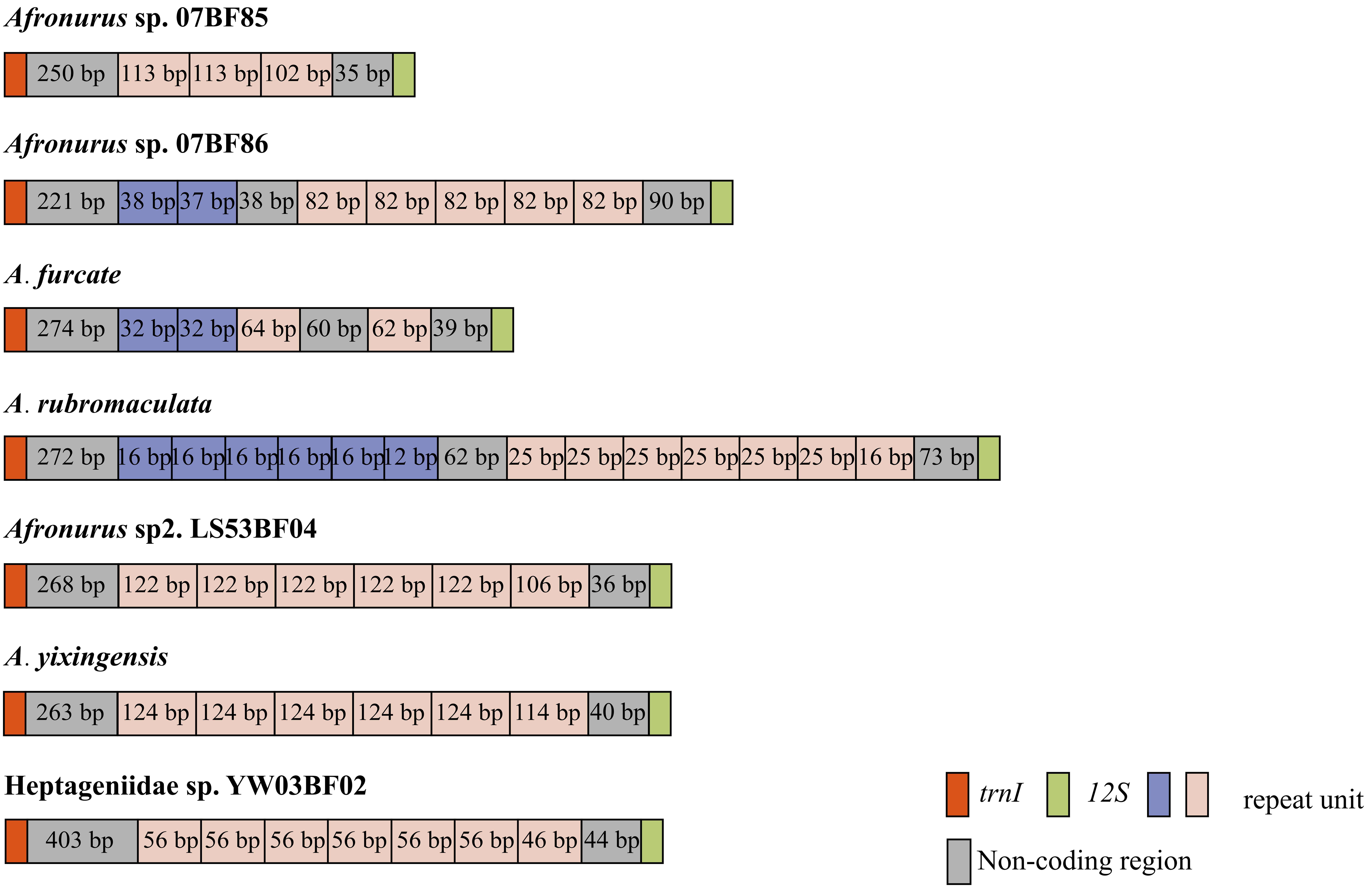

Afronurus sp. 07BF85
250 bp
113 bp
113 bp
102 bp
35 bp
Afronurus sp. 07BF86
221 bp
38 bp
37 bp
38 bp
82 bp
82 bp
82 bp
82 bp
82 bp
90 bp
A. furcate
274 bp
32 bp
32 bp
64 bp
60 bp
62 bp
39 bp
A. rubromaculata
272 bp
16 bp
16 bp
16 bp
16 bp
16 bp
12 bp
62 bp
25 bp
25 bp
25 bp
25 bp
25 bp
25 bp
16 bp
73 bp
Afronurus sp2. LS53BF04
268 bp
122 bp
122 bp
122 bp
122 bp
122 bp
106 bp
36 bp
A. yixingensis
263 bp
124 bp
124 bp
124 bp
124 bp
124 bp
114 bp
40 bp
Heptageniidae sp. YW03BF02
403 bp
56 bp
56 bp
56 bp
56 bp
56 bp
56 bp
46 bp
44 bp
trnI
12S
repeat unit
Non-coding region
