# Supplementary material for: Insight into the Phylogenetic Relationships among Three Subfamilies within Heptageniidae (Insecta: Ephemeroptera) along with Low-Temperature Selection Pressure Analyses Using Mitogenomes
Source: Insects. 2021 Jul 19;12(7):656. doi: 10.3390/insects12070656 (PMC8307263; doi:10.3390/insects12070656)
Supplement: Supplementary file 1 [file insects-12-00656-s001.zip › Figure S2. trnM alignment.pdf]

```
trnM1       1  GGAGAGGTAAGCTAAGGT--AAGCTAACAGGTTCATACCCTGTCTATGGAGAATACTCCC
trnM2       1  AGAAAGGTAAGCTAAATTTAAAGCTCATAGGTTCATACCCTATTTATGGT--TACTCCTC
consensus   1  .**.***********. *..***** * *************.* ***** ..     * *

trnM1      59  CTCTCTA
trnM2      59  TTTCTA-
consensus  61   *    .
```

A. *Afronurus* sp. 07BF85

```
trnM1       1  GGGAAGGTAAGCTAAATT-AAGCTAACAGGTTCATACCCTGTGTATGGAGGGTACTCCCC
trnM2       1  AGAAAGGTAAGCTAAATTCAAGCTCATAGGTTCATACCCTATTTATGGTA---ACTCCTC
consensus   1  .*.***************.***** * *************.* ***** ....***** *

trnM1      60  TTCCTA
trnM2      58  TTTCTG
consensus  61  ** **.
```

B. *Afronurus* sp. 07BF86

```
trnM1       1  GGAGAGGTAAGCTAAGGT--AAGCTAACAGGTTCATACCCTGTGTATGGAGTGTACTCCC
trnM2       1  AGAAAGGTAAGCTAAATTCTAAGCTCATAGGTTCATACCCTATTTATGGT---TACTCCT
consensus   1  .**.***********. *..***** * *************.* ***** ...******

trnM1      59  CTCTCTA
trnM2      58  CTTTCTA
consensus  61  ** ****
```

C. *Afronurus* sp. 07BF96

```
trnM1       1  GGAAAGGTAAGCTAAGACTAAGCTAACAGGTTCATACCCTGTGTATGGAGACTACTCCCC
trnM2       1  AGAAAGGTAAGCTAAACTAAAGCTCATAGGTTCATACCCTATTTATGGATACT-CTCCTC
consensus   1  .**************.   ***** * *************.* ****** ***.**** *

trnM1      61  TCTCTA
trnM2      60  TTTCTA
consensus  61  * ****
```

D. *A. furcata*

```
trnM1       1  GGAAAGGTAAGCTAAGGCTAAGCTAACAGGTTCATACCCTGTGTATGGAGACTACTCCCC
trnM2       1  AGAAAGGTAAGCTAAACTAAAGCTCATAGGTTCATACCCTATTTATGGATACT-CTCCTC
consensus   1  .**************.   ***** * *************.* ****** ***.**** *

trnM1      61  TCTCTA
trnM2      60  TTTCTA
consensus  61  * ****
```

E. *A. rubromaculata*

```
trnM1       1  GGAGAGGTAAGCTAAGA-TTAAGCTAATAGGTTCATACCCTATGTATGGAGATTTCTCCC
trnM2       1  AGAAAGATAAGCTAAGTCTTAAGCTAATAGGTTCATACCCTATCTATGG---TTACCCCT
consensus   1  .**.**.********* .*********************** *****...** * **

trnM1      60  CTCTCTA
trnM2      58  CTTTCTA
consensus  61  ** ****
```

F. *Afronurus* sp1. YW01BF06

```
trnM1       1  GGAAAGGTAAGCTAAATTTAAGCTCACAGGTTCATACCCTGTTTATGGAGAGTTCTCCCC
trnM2       1  AGAAAGGTAAGCTAAATTTAAGCTCACAGGTTCATACCCTGTTTATGGT--TTTCCCCTC
consensus   1  .*********************************************** .. *** ** *

trnM1      61  TTTCTA
trnM2      59  TTTCTA
consensus  61  ******
```

G. *Afronurus* sp2. LS53BF04

```
trnM1       1  GAAGAGATAAGCTAAAGTT--AAGCTAATAGGTTCATACCCTATGTATGGAGACTACTCC
trnM2       1  AGAAAGGTAAGCTAAGTTCCCAAGCTCATAGGTTCATACCCTATTTATGGTTA--ATTCC
consensus   1  ..*.**.********. * ..***** ***************** *****  *..* ***

trnM1      59  TCTCTTTA
trnM2      59  TCTTTCTA
consensus  61  *** * **
```

H. *A. yixingensis*

```
trnM1       1  AGGAAGATAAGCTAAGTATAAGCTCGTAGGTTCATACCCTATTTATGGAGATTGCTCCTC
trnM2       1  GGGAAGATAAGCTAAGTATAAGCTCGTAGGTTCATACCCTATTTATGGAGATTGCTCCTC
consensus   1  .***********************************************************

trnM1      61  TTTCTA
trnM2      61  TTTCCA
consensus  61  **** *
```

I. *E. dayongensis*

```
trnM1       1  GGAAAGATAAGCTAATTGTAAGCTTATAGGTTCATACCCTGTCTATGGAGAATACTCCTC
trnM2       1  AAAGAAATAAGCTAAGTCTAAGCTAGTAGGCTCATACCCTAACTATGGTTCCAACCTTTC
consensus   1  ..*.*.********* * ****** .**** *********. ******     **   **

trnM1      61  TTTCTA
trnM2      61  TTTA--
consensus  61  *** ..
```

J. *Epeorus* sp. LA03FY06

```
trnM1       1  GGAAAGGTAAGCTAAGTGTAAGCTCATAGGTTCATACCCTATTTATGGAGTCTTCTCCCC
trnM2       1  AGAAAGGTAAGCTAAGTGTAAGCTCATAGGTTCATACCCTATTTATGG---CATCCCCTC
consensus   1  .***********************************************...* ** ** *

trnM1      61  TTTCTA
trnM2      58  TTTCTA
consensus  61  ******
```

K. Heptageniidae sp. YW03BF02

```
trnM1       1  GGAAAGATAAGC---TAAGTTAAGCTAATAGGTTCATACCCTATGTATGGAGATCCCTCC
trnM2       1  AGTTAGGTAGGCCATTAAATTGAGCTAGTAGGGTTATATCCTACATA--GGGTTGCTTCC
consensus   1  .*  **.**.**...***.**.*****.**** * *** **** .**..*.* * * ***

trnM1      58  TCTTTCTA
trnM2      59  TCTTTCTA
consensus  61  ********
```

L. *L. aphrodite*
