# Supplementary material for: Insight into the Phylogenetic Relationships among Three Subfamilies within Heptageniidae (Insecta: Ephemeroptera) along with Low-Temperature Selection Pressure Analyses Using Mitogenomes
Source: Insects. 2021 Jul 19;12(7):656. doi: 10.3390/insects12070656 (PMC8307263; doi:10.3390/insects12070656)
Supplement: Supplementary file 1 [file insects-12-00656-s001.zip › Figure S3. stem loop.pdf]

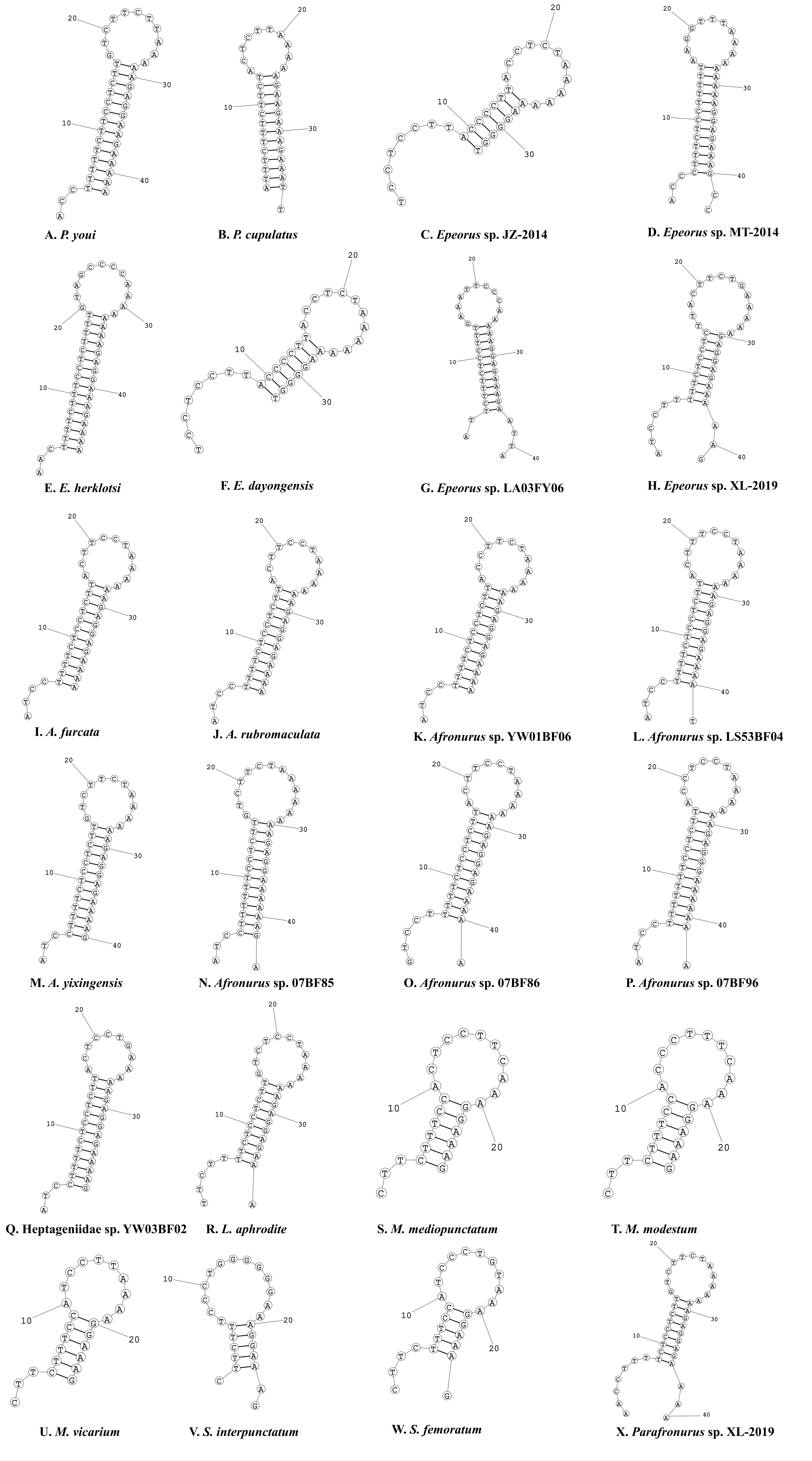
A. P. youi
B. P. cupulatus
C. Epeorus sp. JZ-2014
D. Epeorus sp. MT-2014
E. E. herklotsi
F. E. dayongensis
G. Epeorus sp. LA03FY06
H. Epeorus sp. XL-2019
I. A. furcata
J. A. rubromaculata
K. Afronurus sp. YW01BF06
L. Afronurus sp. LS53BF04
M. A. yixingensis
N. Afronurus sp. 07BF85
O. Afronurus sp. 07BF86
P. Afronurus sp. 07BF96
Q. Heptageniidae sp. YW03BF02
R. L. aphrodite
S. M. mediopunctatum
T. M. modestum
U. M. vicarium
V. S. interpunctatum
W. S. femoratum
X. Parafronurus sp. XL-2019
