# Supplementary material for: Insight into the Phylogenetic Relationships among Three Subfamilies within Heptageniidae (Insecta: Ephemeroptera) along with Low-Temperature Selection Pressure Analyses Using Mitogenomes
Source: Insects. 2021 Jul 19;12(7):656. doi: 10.3390/insects12070656 (PMC8307263; doi:10.3390/insects12070656)
Supplement: Supplementary file 1 [file insects-12-00656-s001.zip › Figure S4 A-NCR-R.pdf]

```
NCR        1 ATCCTTTTCTCCTCTTACTTCCTAAAAAAGAGGAGAAAA
CR         1 AGGTTTTTTTTCTCCTAACCTCTAAGAAGGACCTATATA
consensus  1 *   **** * *** **    ****.**.**   . * *
```

A. *A. furcate*

```
NCR        1 ATCCTTTTCTCCTCTTACTTCCTAAAAAAGAGGAGAAAA
CR         1 AGGTTTTTTTTCTCCTAACCTCTAAGAAGGACCTATATA
consensus  1 *   **** * *** **    ****.**.**   . * *
```

B. *A. rubromaculata*

```
NCR        1 ATCCTTTTCTCCTCTTACCTTCTAAAAAAGAGGAGAAAA
CR         1 AGGTTTTTTTTCCCTTAACCTCTAAGAAGGATCAATATT
consensus  1 *   **** * * **** * *****.**.**  *. *
```

C. *Afronurus* sp1. YW01BF06

```
NCR        1 ATCCTTTTCTCCTCTTACTTTCCTAAAAAAGAGGAGAAAAT
CR         1 AGGTTTTTTTTATCCTAATCTC-TAAGAAGGATCAATATAT
consensus  1 *   **** *  ** ** * **.***.**.**  *. * **
```

D. *Afronurus* sp2. LS53BF04

```
NCR        1 ATCCTTTTCTCCTCTTGTCTTCTAAAAAAGAGGAGAAAAG
CR         1 AGGTTTTTTTTCTCCTAACCTCTAAGAAGGACCAATATAT
consensus  1 *   **** * *** *. * *****.**.**  *. * *
```

E. *A. yixingensis*

```
NCR        1 ATCCTTTTTTCCTCTTGTCTTCTAAAAAAAAGAGGAAAAAAGA
CR         1 AGGTTTTTTTTCTTCTAACCTCTAAGAAGGATTAATATATATA
consensus  1 *   ****** **  *. * *****.**..*  .. * * * *
```

F. *Afronurus* sp. 07BF85

```
NCR        1 GTCCTTTTTCTCCTCTTACTTCCTAAAAAAGAGGAGAAAAA
CR         1 AGGTTTTTTTTACTCCTAACCTCTAAGAAGGATTAATATAT
consensus  1 .   ***** * *** **    ****.**.**  *. * *
```

G. *Afronurus* sp. 07BF86

```
NCR        1 ATCCTTTTTTTCCTCTTACCTCCTAAAAAAGAGGGAAAAAAA
CR         1 AGGTTTTTTTTCTTCTAACCTC-TAAGAAGGATTAATATATA
consensus  1 *   ******** *** *****.***.**.**  .* * * *
```

H. *Afronurus* sp. 07BF96

```
NCR        1 TCCTCCTTACCCCTTACCTCTAAAAA-AAGGGGT
CR         1 TGTTCCATACCCCAAATTCTTATCAATAAACAAT
consensus  1 *  *** ******  *    **  **.**. ..*
```

I. *Epeorus* sp. JZ-2014

```
NCR        1 ACCCT--TTCTCCTTTTTAAGGTTTAAAAAAAAAGGAGAAAGCC
CR         1 ACCCTGTTCCTGCATTTAAAAAGTTACTAGCAGAAAAGTTTGTA
consensus  1 *****..* ** * *** **.. ***  *. *.*..**   *
```

J. *Epeorus* sp. MT-2014

```
NCR         1  AACTTTTCTTTCCTCTTTTGTAGCCCCAAAAAAAGAGGAAAGAAAA
CR          1  TATATGTATAAACCCAGTTATAACCCTCAAAAAAACCTTAAATAACA
consensus   1   *  * * *   * *  **.**.***  *******    *** ** *
```

K. *E. herklotsi*

```
NCR         1  TCCTCCTTACCCCTTACCT-CTAAAAAAAGGGGT
CR          1  ACGTTATTATAACACACTTACTAGATAAAGACAT
consensus   1   * *  ***   *  ** *.***.* ****.  .*
```

L. *E. dayongensis*

```
NCR         1  ATTCTTTCTCCTTTGAAATTCCCAAAAAGGAGAAAGAATTA
CR          1  ATGATATTTCATTGGGTATTATTAAATAATATGATTGATTA
consensus   1  **  * * ** ** *. ***   *** *. * .*  .****
```

M. *Epeorus* sp. LA03FY06

```
NCR         1  ATCCTTTTTCTCCTCTTACTTCTGAAAAAGAGGAGAAAAAG
CR          1  AAGCATTTCCTCCTCT-GCTTCTTCCAAAGAGGAGGA----
consensus   1  *  * *** *******..*****   *********.*....
```

N. *Epeorus* sp. XL-2019

```
NCR         1  ATCCTTTTCTCCTCTTACTCCTGAAAAAGAGGAGAAAAG
CR          1  AGCATTTCCTCCTCTGTCTCTCCCAAA-GAGGAGGA---
consensus   1  * * *** *******  ***    ***.******.*...
```

O. Heptageniidae sp. YW03BF02

```
NCR         1  TTCTTTTCTCCTCTTGTCTCCTAAAAAAGAGGAGAAA
CR          1  TTCATTTCTCCTCTTGTCTCCTAAAAA-GAGGAGAAA
consensus   1  *** ***********************.*********
```

P. *L. Aphrodite*

```
NCR         1  CTTCTTTCCACTCCTTCAAAGGAAAG
CR          1  CCTCTTTCTAATTTCTGGAGATAATA
consensus   1  * ****** * *   * .*.. ** .
```

Q. *M. mediopunctatum*

```
NCR         1  CTTCTTTCCACCCTTTCAAAGGAAAG
CR          1  TACGTTTCTACCCATCTAATAAAAAG
consensus   1      **** **** *  ** ..****
```

R. *M. modestum*

```
NCR         1  CTTCTTTCCATCCTTAAAAGGAAAG
CR          1  CCTCTTTCAAATCTCCGGAGATAAT
consensus   1  * ****** *  **  ..**. **
```

S. *M. vicarium*

```
NCR         1  ACCTTTTTCTTCCTCTTGTCTTCTTAAAAAGAGGAAGAAAAA
CR          1  AAGCATTTTCCCCTCTTGTCTTCTTAAAAAGAGGGGAA----
consensus   1  *    ***   ***********************...*....
```

T. *P. youi*

```
NCR         1 ATTTCTT--TCTTCTACTCTTAAAAAGAAGAAAGAAATT
CR          1 ATTCCTTCTTCTTCCAGAAATAAAAGGAAGAAGAAGA--
consensus   1 *** ***..***** *    *****.******..*.*..
```

U. *P. cupulatus*

```
NCR         1 AACCTTTTCTCCTCTTGTCTTCTAAAAAAAGAGGAGAAAA
CR          1 AGGTTTTTTTTCTCTTAACCTCTAAGAAGGATCAATATTT
consensus   1 *.  **** * *****. * *****.**...  .* *
```

V. *Parafronurus* sp. XL-2019

```
NCR         1 CTTCTTTCCCTGGGGGGAAAGGAAAG
CR          1 ATCCTTTACCGAGGGGGTCATCAAAT
consensus   1  * **** ** .*****  *  ***
```

W. *S. interpunctatum*

```
NCR         1 CTTCTTTCCA-TCCCTGTAAAGGAAAG
CR          1 CCTCTTTCAAATCTCCGGAGATAATAG
consensus   1 * ****** *.** * * *.* .* **
```

X. *S. femoratum*
