# Supplementary material for: Insight into the Phylogenetic Relationships among Three Subfamilies within Heptageniidae (Insecta: Ephemeroptera) along with Low-Temperature Selection Pressure Analyses Using Mitogenomes
Source: Insects. 2021 Jul 19;12(7):656. doi: 10.3390/insects12070656 (PMC8307263; doi:10.3390/insects12070656)
Supplement: Supplementary file 1 [file insects-12-00656-s001.zip › Figure S5. protein.pdf]

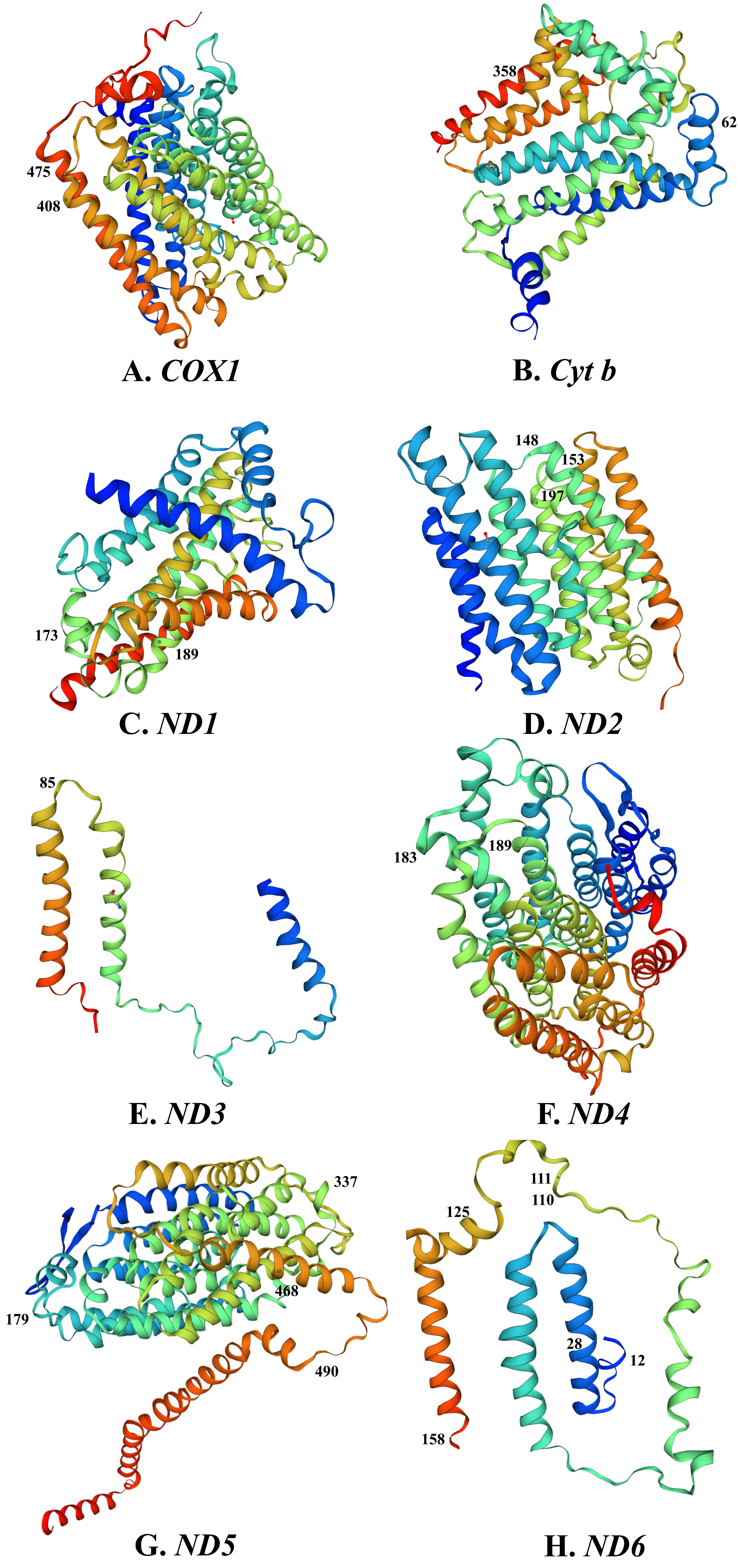

Note. The 318 and 341 amino acid sites at *ND2*, the 25 and 29 amino acid sites at *ND5*, along with the 3 amino acid site at *ND6* were not predicted due to the lack of corresponding template sequences.
