# Supplementary material for: Insight into the Phylogenetic Relationships among Three Subfamilies within Heptageniidae (Insecta: Ephemeroptera) along with Low-Temperature Selection Pressure Analyses Using Mitogenomes
Source: Insects. 2021 Jul 19;12(7):656. doi: 10.3390/insects12070656 (PMC8307263; doi:10.3390/insects12070656)
Supplement: Supplementary file 1 [file insects-12-00656-s001.zip › Figure S6. S1-NCR-ND1.pdf]

Afronurus sp. 07BF85	1	TACTTAAAAAGTTCAG
Afronurus sp. 07BF86	1	TACTTAAAAAGTTCAG
P. youi	1	TACTTAAAAAGTTCAA
Epeorus sp. XL-2019	1	TACTTAAAAAGTTCAA
Epeorus sp. MT-2014	1	TACTTAAAAAGTTCAA
Epeorus sp. JZ-2014	1	TACTTAAAAAGTTCAA
E. herklotsi	1	TACTTAAAAAGTTCAA
S. femoratum	1	TACTTAAAAAGTTCAA
S. interpunctatum	1	TACTTAAAAAGTTCAA
M. vicarium	1	TACTTAAAAAGTTCAA
M. modestum	1	TACTTAAAAAGTTCAA
M. mediopunctatum	1	TACTTAAAAAGTTCAA
Epeorus sp. LA03FY06	1	TACTTAAAAAGTTCAA
E. dayongensis	1	TACTTAAAAAGTTCAA
A. yixingensis	1	TACTTAAAAAGTTCAA
A. rubromaculata	1	TACTTAAAAAGTTCAA
Afronurus sp. 07BF96	1	TACTTAAAAAGTTCAA
P. cupulatus	1	TACTTAAAAAAGTCAA
Afronurus sp2. LS53BF04	1	TACTTAAAAAATTCAA
Afronurus sp1. YW01BF06	1	TACTTAAAAAATTCAA
L. aphrodite	1	TACTTAAAAAATTCAA
Heptageniidae sp. YW03BF02	1	TACTTAAAAAATTCAA
A. furcata	1	TACTTAAAAAATTCAA
Parafronurus sp. XL-2019	1	TACTTAAAAAATTCAA
consensus	1	**********..***.

NCR located between *trnS2* and *ND1*
