# Supplementary material for: Insight into the Phylogenetic Relationships among Three Subfamilies within Heptageniidae (Insecta: Ephemeroptera) along with Low-Temperature Selection Pressure Analyses Using Mitogenomes
Source: Insects. 2021 Jul 19;12(7):656. doi: 10.3390/insects12070656 (PMC8307263; doi:10.3390/insects12070656)
Supplement: Supplementary file 1 [file insects-12-00656-s001.zip › Storey edit - Xiaodong Xu 20210712 with trace(1).pdf]

insects

MDPI

Article

Insight into the Phylogenetic Relationships among Three Subfamilies within Heptageniidae (Insecta: Ephemeroptera) along with Low-temperature Selection Pressure Analyses Using Mitogenomes

Commented [KS1]: We suggest this title change that eliminates 2 hypenated words that made the title look awkward.

Xiao-Dong Xu[a#], Jia-Yin Guan[a#], Zi-Yi Zhang[a], Yu-Rou Cao[a], Yin-Yin Cai[a], Kenneth B. Storey[b], Dan-Na Yu[a,c*] and Jia-Yong Zhang[a,c*]

[a]College of Chemistry and Life Science, Zhejiang Normal University, Jinhua, Zhejiang Province, China;
[b]Department of Biology, Carleton University, Ottawa, Canada; [c]Key Lab of Wildlife Biotechnology, Conservation and Utilization of Zhejiang Province, Zhejiang Normal University, Jinhua, Zhejiang Province, China
[#]These authors contributed to the work equally and should be regarded as co-first authors.
* Correspondence: DN Yu: ydn@zjnu.cn; JY Zhang: zhang3599533@163.com or zhangjiayong@zjnu.cn

Simple Summary: The Ephemeroptera is an ancient lineage of insects, among which the Heptageniidae is one of the most species-rich families although its phylogenetic relationships have been controversial. The mitogenomes of Heptageniidae were found gene rearrangements of CR-*I-M-Q-M-ND2* and a conserved intergenic gap between *trnA* and *trnR*. Thus, 15 complete and two nearly complete mitogenomes of Heptageniidae were used to explore mitogenome structures and clarify the disputes of phylogenetic relationships among Heptageniidae. Additionally, the Heptageniidae samples collected from habitats with significant temperature differences were applied to investigate the adaptive evolution of mitochondrial PCGs under low temperature stress.

Abstract: We determined 15 complete and two nearly complete mitogenomes of Heptageniidae belonging to three subfamilies (Heptageniinae, Rhithrogeninae and Ecdyonurinae) and six genera (*Afronurus*, *Epeorus*, *Leucrocuta*, *Maccaffertium*, *Stenacron* and *Stenonema*). Species of Rhithrogeninae and Ecdyonurinae have the same gene rearrangement of CR-*I-M-Q-M-ND2*, whereas a novel gene rearrangement of CR-*I-M-Q*-NCR-*ND2* was found in Heptageniinae. Non-coding regions (NCRs) of 25-47 bp located between *trnA* and *trnR* were observed in all mayflies of Heptageniidae, which may be a synapomorphy for Heptageniidae. Both the BI and ML phylogenetic analyses supported the monophyly of Heptageniidae and its subfamilies (Heptageniinae, Rhithrogeninae and Ecdyonurinae). The phylogenetic results combined with gene rearrangements and NCR locations confirmed the relationship of the subfamilies as (Heptageniinae + (Rhithrogeninae + Ecdyonurinae)). To assess the effects of low temperature stress on Heptageniidae species from Ottawa, Canada, we found 27 positive selection sites in 8 protein-coding genes (PCGs) using the branch-site model. The selection pressure analyses suggested mitochondrial PCGs underwent positive selection to meet the energy requirements under low temperature stress.

Keywords: Heptageniidae; Mitochondrial genome; Gene rearrangement; Phylogenetic relationship; Non-coding region (NCR), Selective stress analysis

1. Introduction

As a primitive group of winged insects, Ephemeroptera is comprised of 40 families, 460 genera and 3,700 species [1-3]. Ephemeroptera is characterized by multiple ancestral signs including extra appendages (seven pairs of gills on larvae along with the forceps and long tails of adults), unique prometamorphosis development pattern and wings that

Commented [KS2]: The plural of larva is larvae. It was a Latin word originally.

do not fold flat over the abdomen, which have been intensely studied in phylogeny and historical processes [4-6]. As one of the most species-rich families among Ephemeroptera, Heptageniidae consists of 3 subfamilies (Ecdyonurinae, Heptageniinae, and Rhithrogeninae), 37 genera, and 606 described species [1,3,7]. Much effort has been made to figure out the taxonomy and phylogeny of Ephemeroptera using morphology features, molecular proofs and combined data [8-12]. Nonetheless, the phylogenetic relationships within Heptageniidae remain controversial [7,13]. The phylogenetic systems of both McCafferty [9] and Kluge [10,11] supported Heptageniidae as belonging to Branchitergalia (Heptagenioidea) with a close relationship to Isonychiidae. The phylogenetic relationship developed using combined data of morphological characters and several nuclear genes by Ogden et al. [12] was different from the former hypotheses, and supported Heptageniidae as a monophyletic group but its phylogenetic position still remained uncertain. According to the newly published study by Ogden et al. [15], the phylogenetic results supported Heptageniidae as s sister group to Isonychiidae using over 440 targeted genomic protein coding regions (exons). In addition, the internal phylogenetic relationships within Heptageniidae were divided into three subfamilies (Heptageniinae, Rhithrogeninae and Ecdyonurinae) and their relationship was shown as (Ecdyonurinae + (Heptageniinae + Rhithrogeninae)) by Wang & McCafferty [13] and Webb & McCafferty [7]. By contrast, the phylogenetic analysiss was presented as (Rhithrogenidae + (Ecdyonurinae + Heptageniinae)) by Ogden et al. [15]. In addition, the genera *Stenonema* was redefined to include *Maccaffertium* via two mitochondrial genes (*COX1* and *16S rRNA*) and two nuclear genes (*Wingless* (*Wg*) and *histone H3*) by Zembrzuski & Anderson [16].

The typical mitochondrial genome (mitogenome) of insects is a 14-20 kb double-stranded circular piece of DNA [17-19]. It encodes 37 genes including 13 protein-coding genes (PCGs), two ribosomal RNAs (rRNAs, *16S rRNA* and *12S rRNA*), 22 transfer RNAs (tRNAs), and the A+T-rich region (control region, CR). Since the mitogenome has features like rapid evolution rates, small genome sizes, relatively low recombination and maternal inheritance, it is considered to be an excellent molecular marker for studies in phylogeny, evolution and comparative genomics [18,20-22]. Although most insect mitogenomes are conservative, gene rearrangements and long non-coding regions (NCRs) have been variously reported in Coleoptera, Hemiptera, Lepidoptera, Mantodea, Orthoptera, Thysanoptera, etc. [19,23-31]. According to published reports, gene rearrangements of tRNA genes including duplication, translocation and pseudogenization were mainly concentrated in the regions of CR-*I-Q-M-ND2*, *COX1-K-D-ATP8* and *ND3-A-R-N-S-E-F-ND5* [31,32].

Within the order Ephemeroptera, most species retain the same 37 genes as the hypothesized ancestral mitogenome of insects except for Siphluriscidae, Baetidae, Leptophlebiidae, Ephemerellidae and Heptageniidae [32-41]. The mitogenome of *Siphluriscus chinensis* (Siphluriscidae) encoded an extra *trnK* located between *trnS* and *trnE* in the minor coding strand [36]. The *trnC* and *trnY* in *Alainites yixiani* (Baetidae) translocated from a position between *trnW* and *COX1* into the gene cluster of *I-Q-M* and the gene order was rearranged as *I-C-Q-Y-M*. Furthermore, one copy of inversion and translocation of *trnI* was detected in *Ephemerella* sp. Yunnan-2018, *Ephemerella* sp. MT-2014, *Serratella zapekinae*, *Serratella* sp. Liaoning-2019 and *Serratella* sp. Yunnan-2018 along with three duplicate copies of inversion and translocation of *trnI* in *Torleya grandipennis* and *T. tumiforceps* (Ephemerellidae) [32]. The *trnA* and *trnR* genes switched positions in *Habrophlebiodes zijinensis* (Leptophlebiidae) resulting in a gene arrangement *R-A-N-S-E-F*. Within the family Heptageniidae, an extra *trnM* was observed in the location between *trnQ* and *ND2*, thus the gene arrangement was arranged as *I-M-Q-M* in *Epeorus herklotsi*, *Epeorus* sp. JZ-2014, *Epeorus* sp. MT-2014 and *Parafronurus youi* [33-35]. Surprisingly, no gene rearrangements were found in *Paegniodes cupulatus* (Heptageniidae), showing that its gene order was conservative and different from other Heptageniidae species.

Despite the fact that mitogenomes are generally considered to be under neutral or nearly neutral selection [42], several studies have pointed out that positive selection acted on mitochondrial PCGs linked to environmental adaptations [43-45]. In this way, as a

potential target associated with energy metabolism under environmental selection pressure, the mitogenome may be suitable for studying positive selection or natural selection. Based on the mitochondrial PCGs of flying and flightless grasshoppers, a significant positive selection was found in several genes including *ND2*, *ND4*, *ND4L*, *ND5*, *ND6*, *ATP8* and *COX3* in flying lineages [45]. Hence, this indicated that positive selection stimulated mitochondrial genes to better suit the energy demands of flight in grasshoppers. Likewise, the mitochondrial PCGs were affected by positive selection from the last common ancestor of Pterygota and flying insects, which illustrated that those mitochondrial PCGs related to energy metabolism had undergone adaptive evolution during the evolution of flight capacity in insects [45]. As aquatic insects, mayflies spend most of their developmental stages in the water. Among various environmental factors, the water temperature was shown to be crucial to the morphology, behavior, growth and life cycle of mayflies [46,47]. Therefore, mayflies were proposed as appropriate models to investigate the adaptive evolution of aquatic insects in a low-temperature environment.

To explore the characteristics of gene rearrangements and the phylogenetic relationship of subfamilies in Heptageniidae, we determined the mitogenomes of seventeen species from all three subfamilies and six genera of Heptageniidae. The phylogenetic relationship within Ephemeroptera was constructed with gene rearrangements and the location of NCRs to clarify the phylogenetic controversies. Moreover, samples of several Heptageniinae (*Maccaffertium*, *Stenacron* and *Stenonema*) and *Leucrocuta* were collected from Ottawa, Canada. The climate there is so cold that the lowest temperature is below 0 °C and water temperature is below 10 °C for eight months of the year [48]. Thus, these mayfly nymphs had to be exposed to low water temperature for a long time. Other samples of *Epeorus* and *Afronurus* were from southern provinces (Zhejiang and Yunnan) of China where the mean annual water temperature was about 24-26 °C. Accordingly, Heptageniidae samples collected from habitats with significant temperature differences were suitable materials to assess adaptive evolution of mitochondrial PCGs under low temperature stress. In brief, our research not only provided a novel insight into the gene rearrangements and phylogenetic relationship within Heptageniidae, but also inquired into the evolutionary mechanisms of aquatic insect mitochondrial PCGs under low temperature stress.

2. Materials and Methods

2.1. Sampling collection and DNA extraction

Six specimens were collected from the Rideau River, Ottawa, Canada in July, 2017. Eleven specimens were collected from Wu River, Jinhua, Zhejiang province, and Chuan River, Jingdong, Yunnan province, China (Table 1). The specimens were all identified based on a combination of nymph morphology and the alignment of *COX1* genes. Because some new species and genus were found in this study, we only identify those species at the genus or family level (Table 1). Samples were stored in 100% ethanol at -40°C in Dr. JY Zhang's lab, College of Life Science and Chemistry, Zhejiang Normal University, China. Our study included seventeen specimens representing all three subfamilies, nine specimens from the subfamily Ecdyonurinae (*Afronurus* and *Leucrocuta*), five specimens from Heptageniinae (*Maccaffertium*, *Stenacron* and *Stenonema*), and two specimens from Rhithrogeninae (*Epeorus*). Total DNA was extracted from legs or half of the whole individual of every species using Ezup Column Animal Genomic DNA Purification Kit (Sangon Biotech Company, Shanghai, China).

2.2. PCR amplification and sequencing

This study used both normal polymerase chain reaction (PCR) and long-and-accurate PCR (LA PCR) methods with Takara Taq or Takara LA Taq DNA polymerase (Takara, Dalian, China). Normal PCR (product length <3,000 bp) or LA PCR (product length >3,000 bp) reaction conditions were as in Gao et al. [49]. The mitogenomes were amplified in 700-

Commented [13]: You don't mention in the text anything about the origin of the specimens, although Table 1 suggests they were newly collected for this study. Please specify
We know the species should be given more detail collection. But we think it was not the our da

Commented [14]: those 17 specimens include 1 Heptageniidae sp. You don't explain why this specimen remains unassigned to a genus, despite the sequencing of its mtDNA. Please specify.

2000 bp short fragments with universal primers according to the method of Simon et al. [50,51] and Zhang et al. [33]. Afterwards, we designed specific primers (Table S1) with Primer Premier 5.0 [52] according to the obtained sequences. All PCR products were obtained in both forward and reverse directions using the primer-walking method and AB13730XL by Sangon Biotech Company (Shanghai, China).

Table 1. Information on specimen sources of the samples used in this study and NCBI Genbank accession numbers.

Subfamily	Species	Specimen No.	Sampling localities	Accession number
Heptageniinae	*Maccaffertium mediopunctatum* (McDunnough, 1926)	03FY33	Ottawa, Canada	MK642302
	Maccaffertium modestum (Banks, 1910)	03FY62	Ottawa, Canada	MK642303
	Maccaffertium vicarium (Walker, 1853)	03FY39	Ottawa, Canada	MK642304
	Stenacron interpunctatum (Say, 1839)	03FY34	Ottawa, Canada	MK642305
	Stenonema femoratum (Say 1823)	03FY36	Ottawa, Canada	MK642306
Ecdyonurinae	*Leucrocuta Aphrodite* (McDunnough, 1926)	03FY51	Ottawa, Canada	MK642301
	Afronurus furcata (Zhou & Zhen, 2003)	08BF03	Zhejiang, China	MK642293
	Afronurus rubromaculata (You, Wu, Gui & Hsu, 1981)	08BF02	Zhejiang, China	MK642294
	Afronurus sp1. YW01BF06	01BF06	Zhejiang, China	MK642295
	Afronurus sp2. LS53BF04	53BF04	Zhejiang, China	MK642296
	Afronurus yixingensis (Wu & You, 1986)	06BF03	Zhejiang, China	MK642297
	Afronurus sp. 07BF85	07BF85	Yunnan, China	MW450876
	Afronurus sp. 07BF86	07BF86	Yunnan, China	MW450877
	Afronurus sp. 07BF96	07BF96	Yunnan, China	MW450878
Rhithrogeninae	*Epeorus dayongensis* (Gui & Zhang, 1992)	18BF01	Zhejiang, China	MK642298
	Epeorus sp. LA03FY06	03FY06	Zhejiang, China	MK642299
/	Heptageniidae sp. YW03BF02	03BF02	Zhejiang, China	MK642300

2.3. Mitogenome annotation and sequence analyses

We inspected and assembled mitochondrial sequences using DNASTAR Package v.7.1 [53]. All tRNA genes and their secondary structures were identified by MITOS (http://mitos.bioinf.uni-leipzig.de/index.py) [54]. Two rRNA genes (*12S* and *16S rRNA*) and thirteen PCGs were determined by alignments with homologous mtDNA sequences from several species in Heptageniidae using Clustal X [55,56]. The nucleotide composition, codon usage, and relative synonymous codon usage (RSCU) were calculated by Mega 7.0 [56]. The GC skews and AT skews were separately calculated using the following formulae: AT skew = (A-T)/(A+T), and GC skew = (G-C)/(G+C) [57]. Ttandem repeats in CRs were detected via Tandem Repeat Finder V 4.09 [58]. The secondary structures of NCRs were found and mapped via RNAstructure Web Servers [59].

2.4. Phylogenetic analyses

Forty-nine species from Ephemeroptera, including fourteen families (Table 2), were used in phylogenetic analyses of Heptageniidae and Ephemeroptera [32-41]. The taxon *Siphluriscus* (Siphluriscidae) was recovered as a sister clade to all other mayflies and, therefore, *S. chinensis* from the family Siphluriscidae was selected as the outgroup [12,36]. Thirteen PCGs of mayfly mitogenomes were used to construct BI and ML phylogenetic trees. The nucleotide sequences of the 13 PCGs were used for DNA alignment by MAFFT v 7.475 [60] and the conserved regions were detected by Gblock 0.91b using default settings [61]. The program PartionFinder 1.1.1 was employed on the basis of Bayesian information criterion (BIC) to identify the best partitioning scheme and substitution model and all twelve partitions were observed (Table S2) [62]. ML analysis was run in RAxML 8.2.0 with a GTRGAMMAI model and branch support for each node was evaluated with 1,000 replicates [63]. BI analysis was performed in MrBayes version 3.2 using a GTR+I+G model. Each of four chains ran for 10 million generations and sampling every 1,000 generations was used for phylogenetic relationship reconstruction [64]. The convergence was evaluated using Tracer version 1.5 and trees from the first 25% of the samples were removed as burn-in during BI analysis.

Table 2. List of Ephemeroptera mitogenomes used to construct phylogenetic trees.

Family	Genus	Species	Length (bp)	GenBank No.	References
Ameletidae	*Ameletus*	*Ameletus* sp. MT-2014	15,141	KM244682	[35]
Baetidae	*Baetis*	*Baetis* sp. PC-2010	14,883	GU936204	Unpublished
	Takobia	*Alainites yixiani*	14,589	GU479735	Unpublished
Caenidae	*Caenis*	*Caenis* sp. JYZ-2018	15,254	MG910499	[33]
		Caenis sp. JYZ-2020	15,392	MN356096	[37]
		Caenis sp. YJ-2009	15,351	GQ502451	Unpublished
Ephemerellidae	*Ephemerella*	*Ephemerella* sp. MT-2014	14,896	KM244691	[31]
		Ephemerella sp. Yunnan-2018	15,256	MT274127	[32]
	Serratella	*Serratella* sp. Liaoning-2019	15,523	MT274128	
		Serratella sp. Yunnan-2018	15,134	MT274129	
		Serratella zapekinae	15,703	MT274130	
	Torleya	*Torleya grandipennis*	15,523	MT274131	
		Torleya tumiforceps	15,330	MT274132	
Ephemeridae	*Ephemera*	*Ephemera orientalis*	16,463	EU591678	Unpublished
		Ephemera sp. XL-2019	15,314	MK951659	[35]
Heptageniidae	*Afronurus*	*Afronurus furcata*	15,420	MK642293	This study
		Afronurus rubromaculata	15,519	MK642294	This study
		Afronurus sp. 07BF85	15,473	MW450876	This study
		Afronurus sp. 07BF86	15,696	MW450877	This study
		Afronurus sp. 07BF96	15,491	MW450878	This study
		Afronurus sp. YW01BF06	15,360	MK642295	This study
		Afronurus sp. LS53BF04	15,866	MK642296	This study
		Afronurus yixingensis	15,883	MK642297	This study
	Epeorus	*Epeorus dayongensis*	15,337	MK642298	This study
		Epeorus herklotsi	15,502	MG870104	[30]
		Epeorus sp. JZ-2014	15,338	KJ493406	Unpublished
		Epeorus sp. MT-2014	15,456	KM244708	[31]
		Epeorus sp. LA03FY06	15,514	MK642299	This study
	Leucrocuta	*Leucrocuta aphrodite*	15,428	MK642301	This study
	Maccaffertium	*Maccaffertium mediopunctatum*	15,319	MK642302	This study
		Maccaffertium modestum	15,324	MK642303	This study
		Maccaffertium vicarium	15,324	MK642304	This study
	Paegniodes	*Paegniodes cupulatus*	15,715	HM004123	[104]
	Parafronurus	*Parafronurus youi*	15,481	EU349015	[29]
	Stenacron	*Stenacron interpunctatum*	15,330	MK642305	This study
	Stenonema	*Stenonema femoratum*	15,332	MK642306	This study
		Heptageniidae sp. YW03BF02	15,663	MK642300	This study
Isonychiidae	*Isonychia*	*Isonychia ignota*	15,105	HM143892	Unpublished
		Isonychia kiangsinensis	15,456	MH119135	[34]
Leptophlebiidae	*Choroterpides*	*Choroterpides apiculata*	15,199	MN807287	[36]
	Habrophlebiodes	*Habrophlebiodes zijinensis*	14,355	GU936203	Unpublished
Potamanthidae	*Potamanthus*	*Potamanthus* sp. MT-2014	14,937	KM244674	[35]
Siphlonuridae	*Siphlonurus*	*Siphlonurus aestivalis*	15,120	MT862395	Unpublished
		Siphlonurus immanis	15,529	FJ606783	Unpublished
		Siphlonurus sp. MT-2014	14,745	KM244684	[31]
Siphluriscidae	*Siphluriscus*	*Siphluriscus chinensis*	16,616	HQ875717	[32]
Teloganodidae	/	Teloganodidae sp.	12,435	KM244703	[31]
			2,817	KM244670	
Vietnamellidae	*Vietnamella*	*Vietnamella dabieshanensis*	15,761	HM067837	Unpublished
		Vietnamella sp. MT-2014	15,043	KM244655	[31]

2.5. Positive selection analysis

The software EasyCodeML [65] was used to evaluate the selective pressure on the PCGs of Heptageniidae mitogenomes. Due to the significantly lower environment temperatures experienced by the Heptageniidae species from Ottawa, Canada, these were selected as the foreground branch to investigate the molecular evolution trends of mitochondrial PCGs under low temperature stress. Both the branch model and the branch-site model were employed to explore whether positive selection occurred on specific branches and specific sites at the branch [66,67]. The branch models were performed under the one-ratio model (M0) presuming that ω was fixed over all of the tree or the two-ratio model presuming that ω in specific branches was different from the rest of the tree, respectively.

Also, the branch-site models were combined with heterogeneous ω across sites and branches, which allows positive selection along specified branches (Model A) and can be compared against a null model (Model A_{null}) that allows neutral evolution and negative selection. Likelihood ratio tests (LRTs) and Bayes Empirical Bayes (BEB) were used to assess these models and evaluate the posterior probability of positive selection sites, respectively. Additionally, information on the structure and function of the positively selected genes was acquired using UniProt [68] and 3D structures of the corresponding proteins were built by SWISS-MODEL Workspace [69].

3. Results

3.1. General features of the mitogenomes

The seventeen mitogenomes of Heptageniidae used in this study ranged in length from 15,319 bp in *Maccaffertium mediopunctatum* (McDunnough, 1926) [70] to 15,883 bp in *Afronurus yixingensis* (Wu & You, 1986) [71] (Tables S3-S4) and encoded 13 PCGs, two rRNA genes, 22 or 23 tRNA genes (containing an extra *trnM* gene in some species), and one CR (Fig. 1). Most of the genes were encoded on the major strand which also called J strand, whereas the minor strand (N strand) carried the remaining genes (4 PCGs and 8 tRNAs). The A+T content, AT-skew and GC-skew of corresponding regions (mitogenomes, PCGs and rRNAs) were separately calculated for each mayfly species and shared conserved characteristics with others (Table 3). The tRNAs of these mayflies all showed the classical cloverleaf secondary structures.

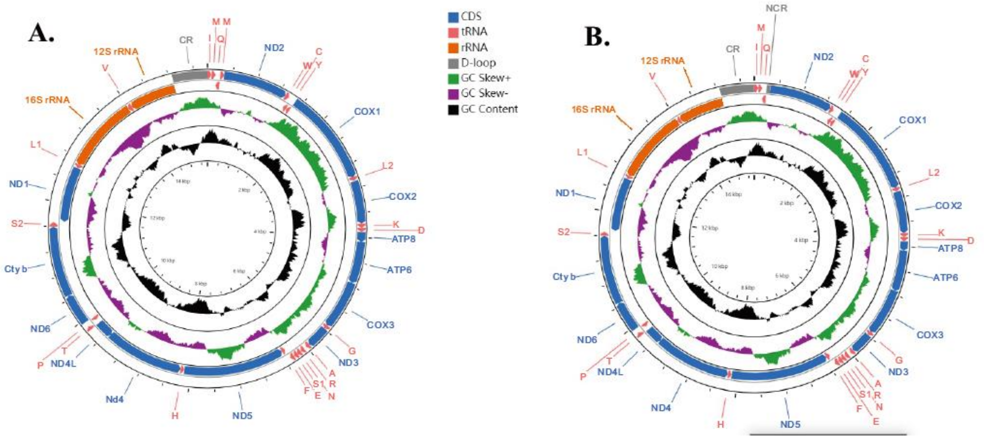

Figure 1. Circular visualization and organization of the complete mitogenome. External genes on the circle are encoded by the positive strand (5'→3') and internal genes are encoded by the negative strand (3'→5'). (A) the mitogenomes in the subfamily Ecdyonurinae (*Afronurus* and *Leucrocuta*) and Rhithrogeninae (*Epeorus*), (B) the mitogenomes in the subfamily Heptageniinae (*Maccaffertium*, *Stenacron* and *Stenonema*).

All these mitochondrial PCGs used conventional invertebrate ATN as start codons, except that *COX1* started with CCG in seven species: *Afronurus* (*A. furcata* (Zhou & Zhen, 2003) [72], *A. rubromaculata* (You, Wu, Gui & Hsu, 1981) [73], *Afronurus* sp. LS53BF04, *Afronurus* sp. YW01BF06, *Afronurus* sp. 07BF85, *Afronurus* sp. 07BF86 and *Afronurus* sp. 07BF96). *ATP8* started with GTG in most mayflies except for *M. mediopunctatum* (McDunnough, 1926) [70], *M. modestum* (Banks, 1910) [13], *M. vicarium* (Walker, 1853) [74], *Stenacron interpunctatum* (Say, 1839) [75], *Stenonema femoratum* (Say 1823) [76] and *Afronurus* sp. YW01BF06. *ND2* started with GTG except for *Epeorus dayongensis* (Gui & Zhang, 1992)

[77], *Epeorus* sp. LA03FY06 and *Afronurus* sp. 07BF96. *ND5* started with GTG except for *Leucrocuta aphrodite* (McDunnough, 1926) [70] and *ND6* started with GTT in *S. interpunctatum*. Typical stop codons TAA and TAG were observed in the majority of PCGs, whereas incomplete stop codons T or TA were assigned in *Cyt b* (*M. mediopunctatum*, *M. modestum*, *M. vicarium* and *S. femoratum*), *COX2* (all species), *ND4* (all species) and *ND5* (all species). The codon number and RSCU in mitochondrial PCGs were conservative among these species (Table S5).

Table 3. Base composition of seventeen mayfly mitochondrial genomes.

**Species name**	Mitogenome-PGC-rRNA-CR	Mitogenome-PGC-rRNA-CR	Mitogenome-PGC-rRNA-CR	Mitogenome-PGC-rRNA-CR	Mitogenome-PGC-rRNA-CR	Mitogenome-PGC-rRNA-CR	Mitogenome-PGC-rRNA-CR	Mitogenome-PGC-rRNA-CR	Mitogenome-PGC-rRNA-CR	Mitogenome-PGC-rRNA-CR	Mitogenome-PGC-rRNA-CR	Mitogenome-PGC-rRNA-CR
	**A+T (%)**				**AT-skew**				**GC-skew**			
Afronurus furcata	64.5	64.4	64.9	56.9	0.005	-0.198	-0.024	0.23	-0.215	-0.004	0.339	-0.16
Afronurus rubromaculata	64.5	64.6	65.0	65.9	0.009	-0.193	-0.025	0.23	-0.218	-0.010	0.327	-0.11
Afronurus sp. 07BF85	63.3	62.8	65.5	60.0	-0.03	-0.210	0.032	0.02	-0.16	-0.012	0.284	0.05
Afronurus sp. 07BF86	64.0	64.4	65.7	54.1	-0.006	-0.191	0.02	0.13	-0.202	-0.017	0.310	0.02
Afronurus sp. 07BF96	62.9	62.4	65.3	/	-0.027	-0.213	0.035	/	-0.16	-0.010	0.288	/
Afronurus sp. YW01BF06	64.6	64.7	64.4	/	0.012	-0.186	0.002	/	-0.225	-0.010	0.317	/
Afronurus sp. LS53BF04	63.3	63.3	64.7	60.1	0.012	-0.198	-0.022	0.16	-0.227	-0.010	0.329	-0.30
Afronurus yixingensis	65.0	65.1	66.0	63.2	0.003	-0.203	-0.002	-0.01	-0.218	-0.004	0.305	-0.21
Epeorus dayongensis	64.8	64.2	66.1	73.6	0	-0.191	0.012	0.04	-0.212	-0.024	0.269	0.01
Epeorus sp. LA03FY06	67.1	66.3	67.6	77.8	-0.014	-0.188	0.02	0.00	-0.24	-0.003	0.307	-0.19
Leucrocuta aphrodite	65	65.0	65.1	65.4	-0.001	-0.187	-0.001	0.02	-0.186	-0.001	0.284	-0.18
Maccaffertium mediopunctatum	61.7	61.5	60.7	65.9	-0.002	-0.190	0.015	0.02	-0.178	-0.045	0.234	-0.35
Maccaffertium modestum	61.3	61.1	60.7	64.0	0.004	-0.191	0.025	0.01	-0.177	-0.038	0.231	-0.33
Maccaffertium vicarium	62.3	62.3	61.8	65.4	0.005	-0.169	0.013	0.00	-0.172	-0.046	0.253	-0.25
Stenacron interpunctatum	59.7	59.5	58.8	61.7	0.021	-0.184	0.007	0.05	-0.19	-0.057	0.266	-0.24
Stenonema femoratum	62.1	61.9	61.1	66.4	0.005	-0.190	-0.011	0.01	-0.166	-0.039	0.234	-0.30
Heptageniidae sp. YW03BF02	64.2	64.1	64.3	68.5	-0.006	-0.191	-0.011	-0.09	-0.183	0.002	0.27	-0.19

The CRs of Heptageniidae mitogenomes ranged from 487 bp to 1,037 bp, with the location between *12S rRNA* and *trnI*. Almost all CRs of these mitogenomes showed the highest A+T content compared to other regions (PCGs, rRNA genes and tRNA genes) except for *A. furcata*, *A. yixingensis*, *Afronurus* sp. 07BF85, *Afronurus* sp. 07BF86 and *Afronurus* sp. LS53BF04. The A+T contents of these CRs ranged from 54.1% in *Afronurus* sp. 07BF86 to 77.8% in *Epeorus* sp. LA03FY06. The AT-skew values of the CRs were a little positive except for *A. yixingensis*, *Epeorus* sp. LA03FY06 and Heptageniidae sp. YW03BF02, whereas the GC-skew was strongly negative except for *Afronurus* sp. 07BF85, *Afronurus* sp. 07BF86 and *E. dayongensis*. Additionally, tandem repeats were detected in the CRs of *Afronurus* sp. 07BF85, *Afronurus* sp. 07BF86, *Afronurus* sp. LS53BF04, *A. furcata*, *A. rubromaculata*, *A. yixingensis* and Heptageniidae sp. YW03BF02 (Fig. S1).

3.2. Gene arrangements and NCRs

Two types of gene rearrangements occurred in the *I-Q-M* tRNA cluster and were found in all seventeen freshly sequenced mitogenomes of Heptageniidae (Fig. 2). The extra *trnM* was observed in the eleven mitogenomes of the subfamily Ecdyonurinae (*Afronurus* species, and *L. aphrodite*) and Rhithrogeninae (*Epeorus* species). As for the location of two *trnM* copies, one was situated between *trnI* and *trnQ* with another between *trnQ* and *trnM*. Thus, their tRNA cluster was shown as *I-M-Q-M*. The two copies of *trnM* genes showed high similarity (>70%) and had the same anti-codon (CAU) in nearly all species except for the second *trnM* (UAU) in *L. aphrodite* (Fig. S2). However, in mitogenomes of the subfamily Heptageniinae (*Maccaffertium* species, *S. interpunctatum* and *S. femoratum*), a translocation of *trnM* was found and the *trnM* gene translocated into the position between *trnI* and *trnQ*. Furthermore, the NCR of 55-57 bp located between *trnQ* and *ND2* was detected in these species but showed low similarity to adjacent genes. Hence, the gene order in the species of Heptageniinae was shown as *I-M-Q*-NCR and this is the first report of this novel gene rearrangement (*I-M-Q*-NCR) among mayfly mitogenomes.

Commented [15]: some details are unclear: the separation between the 3 categories (A+T(%), AT-skew and GC-skew) is not straightforward, as well as the meaning of "Mito". Please improve.

Commented [KS6]: I would add the definition of "Mito" to the title line of the table. I tried to alter the spacing on the table in order to be able to better separate the 3 groups of data columns but it was just too difficult to do within the formatting of this document. You may need to re-fix the table by moving it into Word and send a new Word of Table 3 with the revision. This is the second MDPI paper that we've had this year and both times, it has proven overly difficult as an author to deal with their immediate formatting of the manuscript into a "page proof" sort of style.

Changes that I would make are:

(a) Change the species name column to be left-aligned, not centered, and then you will gain more space between it and the first Mito column. Then drag the first Mito column to the left a bit and the try to increase the space between the 3 groups of columns.

(b) Remove the "s' from PCGs and from RNAs which will help to reduce crowding.

(c) Remove the border lines from underneath the A+T/ AT skew/ GC skew headers and the Mito/PGC/rRNA/CR lines and INSTEAD, use the underline function or the line draw function to place a line underneath each Mito-PGC-rRNA-CR group.

(d) Hence with a greater separation of each of the 3 Mito-PGC-rRNA-CR groups and separate underlining to mark the 3 different groups, this should help readers to see the data better.

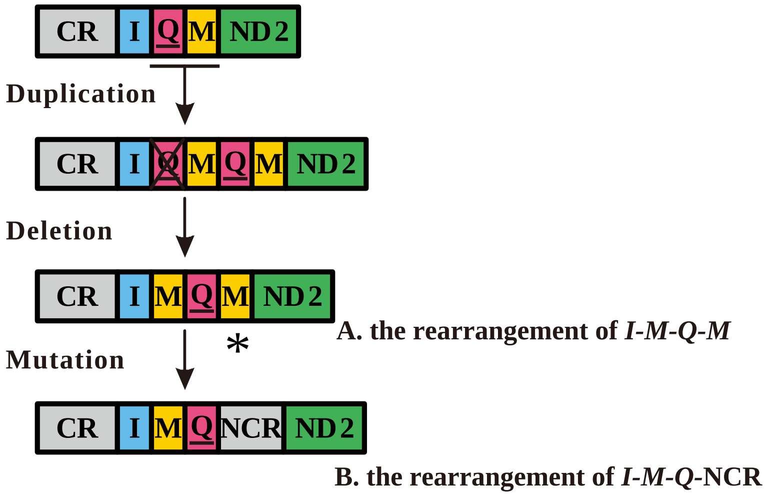

Figure 2. Proposed mechanism of gene rearrangements in the seventeen Heptageniidae mitogenomes. Gene sizes are not drawn to scale. Genes encoded by the L-strand are underlined whereas those without underline are encoded on the H-strand. Different colored boxes represent different genes. The remaining genes and gene orders that were identical to the ancestral insect are left out. Horizontal lines, asterisk symbols and crossed-out symbols represent gene duplications, gene mutations and gene deletions, respectively. (A) the rearrangement of *I-M-Q-M*, (B) the rearrangement of *I-M-Q*-NCR.

The length, number and distribution of the NCRs in these mitogenomes of Heptageniidae were relatively conservative. The number of NCRs in every mayfly species ranged from 7 to 12, whereas the length varied from 1 bp to 57 bp. Excluding the NCRs of short length (<15 bp) and the NCR located between *trnQ* and *ND2* (mentioned above), the NCRs located between *trnA* and *trnR* were observed in all Heptageniidae mitogenomes with lengths ranging from 25 bp (*M. vicarium*) to 47 bp (*E. herklotsi*). Interestingly, the NCRs could be folded as stem-loop secondary structures (Fig. S3) and were highly similar (>70%) to CR based on a comparison among the mitochondrial genomic sequences of most species (Fig. S4). Notwithstanding, the similarity between the NCR and CR was not exactly high (<70%) or the similarity sequence was short (<20 bp) in *A. furcata*, *A. rubromaculata*, *Afronurus* sp.-07BF86, *E. dayongensis*, *Epeorus* sp.-LA03FY06, *M. mediopunctatum*, *M. modestum*, *M. vicarium*, *S. interpunctatum* and *S. femoratum*. We also observed NCRs located between *trnS2* and *ND1* in all mitogenomes of Heptageniidae, which were of 16 bp in length.

3.3. Phylogenetic analyses

The BI and ML phylogenetic relationships showed identical topologies (Fig. 3). However, long-branch attraction (LBA) has been observed in Baetidae (*Baetis* sp. PC-2010 and *Alainites yixiani*) and Teloganodidae sp. MT-2014 and thus their phylogenetic positions still remain uncertain. In general, the monophyly of most families was supported in these phylogenetic trees except for Ephemeridae and Siphlonuridae, but the availability of only one species in Ameletidae, Polymitarcyidae, and Teloganodidae restricted a discussion of their monophyly and phylogenetic relationships.

Commented [KS7]: Editorial staff: After making/accepting changes in the manuscript, would you please adjust the positions of all Figures and Table so that (as much as possible) each figure/table is kept intact on a single page.

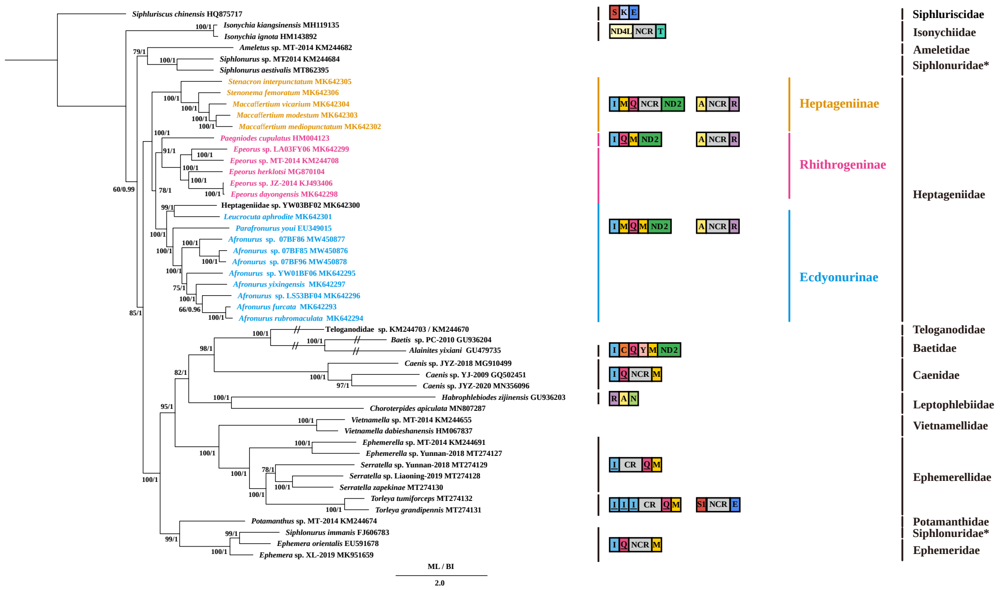

Figure 3. Phylogenetic tree of the relationships among 49 species of Ephemeroptera based on the nucleotide dataset of the 13 mitochondrial PCGs. *Siphluriscus chinensis* was used as the outgroup. The numbers above branches specify bootstrap percentages from ML (left) and posterior probabilities as determined from BI (right). The GenBank accession numbers of all species are shown in the figure. Box images on the right show gene rearrangements and the location of the NCR for the various mayfly species. Genes encoded by the minority strand are underlined and those without underline are encoded by the majority strand. Different colored boxes represent different genes. The remaining genes and gene orders that were identical to the ancestral insect are left out. Gene sizes are not drawn to scale. The asterisks (*) by Siphlonuridae on the far right side mean the separation of these sequences

Within Ephemeroptera, Isonychiidae was a sister group to the other familiesy, based on the phylogenetic topologies. Then, Ameletidae (*Ameletus* sp. MT-2014) and one branch of Siphlonuridae (*Siphlonurus aestivalis* and *Siphlonurus* sp. MT-2014) were found to be a sister group. Heptageniidae was supported as a sister clade to the remaining Ephemeroptera (Baetidae, Caenidae, Ephemerellidae, Ephemeridae, Leptophlebiidae, Potamanthidae, Teloganodidae, and Vietnamellidae). Potamanthidae was the sister clade to (Ephemeridae + *Siphlonurus immanis*), whereas the remaining families formed another large clade. (Ephemerellidae + Vietnamellidae) was supported as a sister clade to (Leptophlebiidae + (Caenidae + (Baetidae + Teloganodidae))).

Concentrating on the phylogenetic relationship within Heptageniidae, the monophyly of three subfamilies (Ecdyonurinae, Heptageniinae and Rhithrogeninae) and the genera *Afronurus*, *Epeorus* and *Maccaffertium* was supported. The branch of Heptageniidae was divided into three clades, shown as follows: (Heptageniinae + (Rhithrogeninae + Ecdyonurinae)). The first branch of Heptageniinae supported (*S. interpunctatum* + (*S. femoratum* + *Maccaffertium* species)). Then *Paegniodes cupulatus* and *Epeorus* species formed the second branch of Rhithrogeninae. The third branch of Ecdyonurinae supported ((Heptageniidae sp. YW03BF02 + *L. aphrodite*) + (*P. youi* + *Afronurus* species)).

Significantly, the phylogenetic relationships coincided with the gene order and the location of the NCRs. The lineage of Ephemerellidae was consistent with rearrangements of the *trnI* gene. The NCRs located between *ND4L* and *trnT* were found in the branch of Isonychiidae along with the NCRs located between *trnQ* and *trnM* in the branch of

Caenidae. Within Heptageniidae, these branches corresponded to different gene arrangements: the gene rearrangement of *I-M-Q*-NCR in Heptageniinae, and *I-M-Q-M* in the remaining species of Rhithrogeninae and Ecdyonurinae except for *I-Q-M* in *P. cupulatus*.

3.4. Positive selection analysis

Based on the branch model, 3,722 amino acid sites were used to analyze selective pressure based on the alignment of 13 PCGs in 22 species of Heptageniidae. The results were as follows: p<0.001, ω_0=0.02021, ω=0.02123<1, illustrating that the foreground branch (the Heptageniinae species from Ottawa, Canada) were not subject to positive selection (Table S6). On the contrary, we observed that 27 amino acid sites were under positive selection (p<0.001, BEB value >0.95) in the analyses of the branch-site models, of which five amino acid sites were under highly positive selection (BEB value >0.99) (Table 4). The 27 positive selection sites corresponding to the mitochondrial PCGs were divided into eight genes, including *COX1* (2 sites), *Cyt b* (2 sites), *ND1* (2 sites), *ND2* (5 sites), *ND3* (1 site), *ND4* (2 sites), *ND5* (6 sites) and *ND6* (7 sites). Accordingly, the mitochondrial complex I was the main protein complex under selective pressure, including 23 positive selection sites. To determine the functional meaning of these positive selection sites, we explored the feature keys of eight positively selected PCGs from low-temperature branches. The majority of the positive selection sites located within or near to the functional domains of the proteins were encoded by these genes; 14 of which were situated in the protein transmembrane domain of the encoding genes with additionally 6 positive selection sites situated in other domains of corresponding genes (Table 5, Fig. S5).

Table 4. Positive selection analysis of mitochondrial protein-coding genes based on the branch-site model.

Tree	Model	Ln L	Estimates of parameters					Model compared	2ΔL	LRT P-value	Positive sites
**ML**	**Model A**	**-121931.169602**	Site class	0	1	2a	2b	Model A vs Model A null	17.1487	0.00004434	682 Q 0.988*，749 S 0.979*，1340 L 0.977*，1636 V 0.954*，1827 E 0.989*，1843 A 0.983*，2118 P 0.995**，2123 F 0.983*，2167 S 0.979*，2288 T 0.983*，2311 T 0.991**，2398 A 0.970*，2613 S 0.962*，2619 M 0.986*，3001 L 0.971*，3005 S 0.964*，3155 S 0.969*，3313 S 0.984*，3444 S 0.989*，3466 H 0.978*，3557 L 0.976*，3566 I 0.996**，3582 C 0.985*，3664 E 0.996**，3665 Q 0.984*，3679 I 0.993**，3712 Q 0.981*
			Proportion	0.9141	0.0377	0.0463	0.0019				
			Background ω	0.0137	1.0000	0.0137	1.0000				
			Foreground ω	0.0137	1.0000	2.7648	2.7648				
	Model A Null	-121939.505555	/								/

Table 5. The features and description of the positive selection sites detected in the mitochondrial PCGs of Heptageniidae species.

Genes	Positive selection sites	Amino acids		BEB value	Feature key*	Description
		Foreground	Background			
COX1	**408**	**N**	**Q/K**	0.988*	Domain	COX1
	475	A	S	0.979*	Domain	COX1
Cyt b	62	T	L	0.977*	Domain	CYTB_NTER
	358	T/I	V/T/I	0.954*	Transmembrane	Helical
ND1	173	Y	E/K/Q	0.989*	/	/
	189	S	A/T	0.983*	Transmembrane	Helical
ND2	148	S/G	P/S	0.995**	Transmembrane	Helical
	153	T/S	F	0.983*	Transmembrane	Helical
	197	N	S/P/N	0.979*	Domain	Proton_antipo_M
	318	G/N	S/L/T/A	0.983*	/	/
	341	S	S/P/T/L	0.991**	/	/
ND3	85	Q	A/N/S/T	0.970*	/	/
ND4	183	S	G/S/K	0.962*	Transmembrane	Helical
	189	G	M/L	0.986*	Transmembrane	Helical
ND5	25	T	L	0.971*	Transmembrane	Helical
	29	L/A	S	0.964*	Transmembrane	Helical
	179	E/H	S/G/T	0.969*	Transmembrane	Helical
	337	S	S/N/I/T	0.984*	Domain	Proton_antipo_M
	468	L	S/V/I/A	0.989*	Transmembrane	Helical
	490	F	N/H/Q/G/S	0.978*	Domain	NADH5_C
ND6	3	T	L/F/M	0.976*	/	/
	12	L	T/I	0.996**	Transmembrane	Helical
	28	I/V	C/S/I/V	0.985*	Transmembrane	Helical
	110	S	E/D	0.996**	/	/
	111	D	Q	0.984*	/	/
	125	P	N/T/I/G	0.993**	Transmembrane	Helical
	158	N	Q/N	0.981*	Transmembrane	Helical

Note. * and ** indicate BEB values of >0.95 and >0.99, respectively.

4. Discussion

4.1. Gene arrangements and NCRs

The typical gene arrangement occurs in most mayfly mitogenomes, except for Siphluriscidae, Baetidae, Leptophlebiidae, Ephemerellidae and Heptageniidae [32-41]. Gene rearrangements in these groups are mainly concentrated in the regions of CR-*I-Q-M-ND2* and *A-R-N-S-E-F*. In our study, the gene rearrangements in the mitogenomes of Heptageniidae were divided into two types: a gene arrangement of *I-M-Q-M* in the subfamily Ecdyonurinae (*Afronurus*, *Parafronurus* and *Leucrocuta*) and Rhithrogeninae (*Epeorus*) and a novel gene arrangement of *I-M-Q*-NCR in the subfamily Heptageniinae (*Maccaffertium*, *Stenacron* and *Stenonema*). Moreover, two copies of *trnM* genes had the same anti-codon (CAU) in almost all species excluding the second *trnM* (UAU) in *L. aphrodite*. The codon AUA is translated as Met in the invertebrate mitochondrial genetic code, like the normal codon AUG, as reported in the fruit fly *Drosophila melanogaster* [78,79]. Therefore, the second *trnM* with anti-codon (UAU) was considered to be functional in *L. aphrodite*. Furthermore, similar gene rearrangements occurring in the region of CR-*I-Q-M-ND2* were also reported in other orders of insects, e.g., *M-I-Q* tRNA cluster in Lepidoptera (*Manduca sexta*) [80], *Q-I-M* in Hemiptera (*Neuroctenus parus*) [81], *I-I-I-I-I-Q-M* in Mantodea (*Schizocephala bicornis*) [31], etc. Consequently, the region of CR-*I-Q-M-ND2* is regarded as a hot spot for gene rearrangements in insects.

The tandem duplication-random loss (TDRL) model [82,83] was proposed and has explained similar gene rearrangements in other insects [81,84]. Therefore, the TDRL model can be reasonably used to explain the gene rearrangements of Heptageniidae (Fig. 2). The region of CR-*I-Q-M-ND2* was presumed to be the original gene arrangement. The mechanisms of gene rearrangement of *I-M-Q-M* was assumed to be as follows: a tandem duplication of *Q-M* happened, followed by random loss of the first *trnQ*, making the gene order as *I-M-Q-M*, as reported in Zhang et al. [33]. As for the gene rearrangement of *I-M-*

Q-NCR, we propose that the tandem duplication of *Q-M* happened, followed by the random loss of the first *trnQ* and the mutation of the second *trnM*. Consequently, the translocation of *trnM* and the NCR located between *trnQ* and *ND2* were observed in the subfamily Heptageniinae (*Maccaffertium*, *Stenacron* and *Stenonema*). Notwithstanding, the mitogenome of *P. cupulatus* showed the typical insect gene order and was different from other mitogenomes of Heptageniidae. The conservative gene order of *P. cupulatus* was proposed to occur as follows: the extra *trnM* between *trnI* and *trnQ* was lost from the ancestral *I-M-Q-M* type and thus formed the *I-Q-M* gene arrangement. Therefore, the gene arrangements among genera of Heptageniidae need further study.

Generally, insect mitogenomes are of high compaction with rare and short NCRs except for the CR [17]. The great majority of mayfly mitogenomes featured short NCR lengths. However, NCRs of 25-47 bp were located between *trnA* and *trnR* and observed in all mitogenomes of Heptageniidae [33-35]. This feature is rarely observed in other mayfly mitogenomes. Thus, this NCR located between *trnA* and *trnR* may be a synapomorphy for Heptageniidae. The NCRs can form stem-loop secondary structures (Fig. S3), which may contribute to the progress of replication slippage and then an increase in duplicate copies [85]. Also, the NCR was proposed as an alternative replication origin for mtDNA [86,87]. As for the occurrence of the NCR, it was inferred to derive mainly from the corresponding CR because of the high similarity between the two (>70%), such as the complete sequence (37 bp) in *L. aphrodite* (similarity 94.59%) and the partial sequence (23 bp) in *P. youi* (similarity 100%) (Fig. S4). Considering the long distance between the NCR and CR, the recombination model may be more suitable to explain NCR production [88,89]. The creation of the NCR was presumed to occur as follows: a fragment containing the CR was cleaved out and then inserted into a location between *trnA* and *trnR*. Although there is a low similarity between the NCR and CR (<70%) or the short similar sequence (<20 bp) in several species (as mentioned in the results), the NCR was proposed to have evolved independently under relaxed selective pressure instead of evolving in concert with the CR [89]. In addition, the short NCR located between *trnS2* and *ND1* was detected in all Heptageniidae mitogenomes, and has also been reported in Ephemeroptera and other insects [23,32-34,49]. Based on the alignments of these NCRs of all Heptageniidae species (Fig. S6), a highly conserved motif of 16 bp (TACTTAAAAARKTCAR) may be the binding site of the transcription termination factor (DmTTF) [90].

4.2. Phylogenetic analyses

Higher-level phylogenetic relationships within Ephemeroptera have not been generally accepted [8-12]. In our results, the BI and ML phylogenetic analyses shared congruent topologies (Fig. 3). *S. chinensis*, the only species of Siphluriscidae, was deemed as the basal group of Ephemeroptera from the study of Ogden et al. [12] and Zhang et al. [33]. The next were Isonychiidae, Ameletidae, and one species of Siphlonuridae, as indicated by our results. The phylogenetic position of Isonychiidae was convincingly supported by Ogden et al. [12] as the primitive clade except for Siphluriscidae and Baetidae from topologies. Then, Heptageniidae was supported as a sister clade to the remaining Ephemeroptera by our results, contrary to the topologies constructed by Kluge [10,11] and McCafferty [8,9], as well as Ogden et al. [15], which suggested that Heptageniidae was sister to Isonychidae based on morphological characteristics and nuclear data, respectively. Based on a comparison of our results and other studies, the phylogenetic position of Heptageniidae is still challenging to determine.

As for Heptageniidae, the monophyly of three subfamilies Ecdyonurinae, Heptageniinae and Rhithrogeninae was supported, consistent with the research of Wang & McCafferty [13] and Webb & McCafferty [7]. Nevertheless, the internal phylogenetic classification within Heptageniidae in our study differed from Wang & McCafferty [13]. In our study, the phylogenetic relationship within Heptageniidae was shown as (Heptageniinae + (Ecdyonurinae + Rhithrogeninae)), opposite to the (Ecdyonurinae + (Heptageniinae + Rhithrogeninae)) presented in Wang & McCafferty [13] and (Rhithrogenidae +

(Ecdyonurinae + Heptageniinae)) in Ogden et al [15]. When the phylogenetic classification was combined with gene rearrangements and NCRs, there was a specific correlation. The gene rearrangement of *I-M-Q*-NCR was concentrated in Heptageniinae and the gene arrangement of *I-M-Q-M* was observed in the remaining species of Rhithrogeninae and Ecdyonurinae except for *I-Q-M* in *P. cupulatus*. These results illustrated that Ecdyonurinae and Rhithrogenina were closely related and the two formed a sister group to Heptageniinae. Phylogenetic relationships highly congruent with gene rearrangements and NCR locations were also reported in other insects [91,92], which suggests that synapomorphic gene rearrangements and NCRs have been forming continuously during evolution and could provide effective phylogenetic information. However, the phylogenetic relationship of the three subfamilies within Heptageniidae was also controversial due to the lack of other evidence in our study. In addition, concerning the taxonomy of *Stenonema*, *Stenacron* and *Maccaffertium*, compared to the research of Zembrzuski & Anderson [16], it is a pity that we could not draw a valid conclusion based on our results because of the lack of sequences for these genera. Further morphological and molecular data are required to demonstrate a more exact phylogenetic relationship among Ephemeroptera.

In fact, the gene arrangement of *I-Q-M* was found only in *P. cupulatus* of Heptageniidae. This was confusing as to whether such a gene arrangement was specific to the genus *Paegniodes* or formed during the random mutation progress. More mayfly mitogenomes are expected to be sequenced, which will help to explore the types of gene arrangements and clear phylogenetic classifications within Heptageniidae.

4.3. Positive selection analyses

Adaptive evolution of mitochondrial genes under environmental pressure is supported by the present studies [43-45]. Environmental temperature significantly influences energy requirements and metabolic adaptation, which is essential to mayflies as aquatic insects [46,47]. Multiple subunits of the mitochondrial complexes associated with oxidative phosphorylation are encoded by mitochondrial genes, with the exception of complex II [93]. In this way, positive selection of mitochondrial genes was proposed to be related to temperature and adaptation to the energy demands of mayflies.

Analysis of the branch model showed that there was no positive selection on the foreground branch. It was proposed that information indicating positive selection was possibly covered by continuous neutral evolution or negative selection at most sites in the gene sequence [94]. According to the branch-site model, 27 positive selection sites were found. It was worth noting that 23 of the positive selection sites were concentrated on the coding sequence of mitochondrial complex I. As the first large protein complex of the respiratory chains, complex I provides the proton power for ATP synthesis during electron transfer from NADH to ubiquinone via the transmembrane proton pump [95-97]. Therefore, complex I is essential for the energy metabolism of cells and drives more than one-third of the total energy production in the mitochondrion [87]. *ND1*-*ND6* subunits are regarded as the minimal assembly of complex I and form the core of the transmembrane region [98], with the *ND2*, *ND4* and *ND5* genes proposed to be main candidates to harbor the proton pump [99]. The importance of complex I and its subunits can explain the reason for more positive selection sites in complex I than in other complexes. In addition, several positive selection sites were also observed in the subunits (*Cyt b* and *COX1*) of complex III and complex IV. *Cyt b* is the main transmembrane subunit of Complex III and exerts a crucial function in ATP production [100]. Also, complex IV has regulatory effects in the electron transport chain and its subunit *COX1* starts the assembly process of complex IV [101]. Moreover, the positive selection sites in the eight PCGs were located in or close to the functional domains based on the structural analysis (Table 5). Consequently, the adaptive changes in amino acids at these positive selection sites, especially in the functional regions, were proposed to affect protein stability or even function [102,103]. On the whole, mitochondrial PCGs are related to energy metabolism and can experience positive selection to cope with energy needs under low temperature stress.

5. Conclusion

Fifteen complete mitogenomes and two nearly complete mitogenomes of Heptageniidae were successfully determined. Gene rearrangements in these mitogenomes of Heptageniidae were divided into two types: one has the commonly reported gene order of *I-M-Q-M* in the subfamily Ecdyonurinae (*Afronurus*, *Parafronurus* and *Leucrocuta*) and Rhithrogeninae (*Epeorus*), whereas the other has a novel gene order of *I-M-Q*-NCR in Heptageniinae (*Maccaffertium*, *Stenacron* and *Stenonema*). These gene rearrangements were explained by the tandem duplication-random loss (TDRL) model. In addition, the NCRs located between *trnA* and *trnR* were found in all Heptageniidae species and inferred to be a synapomorphy for Heptageniidae. The phylogenetic relationships within Ephemeroptera were highly congruent with the gene rearrangements and the location of NCRs, supporting the monophyly of Heptageniidae and its internal phylogenetic relationship (Heptageniinae + (Ecdyonurinae + Rhithrogeninae)). The selection pressure analyses indicated that mitochondrial PCGs of mayflies underwent positive selection to cope with potential energy requirements under low temperature stress.

Supplementary Materials: The following are available online at www.mdpi.com/xxx/s1, Figure S1: title, Table S1: title, Video S1: title. Figure S1. Organization of the repeat regions in the CRs of Heptageniidae species. Figure S2. Alignment between two copies of trnM sequences in Heptageniidae species. Figure S3. The possible secondary structure of the NCRs located between trnA and trnR. Figure S4. Alignment between CRs and the NCRs located between trnA and trnR in Heptageniidae species. Figure S5. Distribution of mutations in the three-dimensional structure of COX1, Cyt b, ND1, ND2, ND3, ND4, ND5 and ND6 genes for the Heptageniidae species from Ottawa, Canada. Figure S6. Alignment of the NCRs located between trnS2 and ND1 in Heptageniidae species. Table S1. Specific primers used to amplify the Heptageniidae mitogenomes. Table S2. The partition schemes and best-fit models selected. Table S3. Location of features in the mitogenomes of the subfamily Ecdyonurinae (Afronurus, Parafronurus and Leucrocuta) and Rhithrogeninae (Epeorus). Table S4. Location of features in the mitogenomes of the subfamily Heptageniinae (Maccaffertium, Stenacron and Stenonema). Table S5. The codon number and relative synonymous codon usage in mitochondrial PCGs. Table S6. Positive selection analysis of mitochondrial PCGs based on the branch model.

Acknowledgements: We would like to thank Huan Zhang for her help in the experiments.

Funding: This work was supported by the Natural Science Foundation of Zhejiang Province (LY18C040004), the College Students' Innovation and Entrepreneurship Project of China (201910345030 and 202010345026) and the College Students' Innovation and Entrepreneurship Project of Zhejiang Province (202010345R128). The funders had no role in study design, data collection and analysis, decision to publish, or preparation of the manuscript.

Author Contributions: Conceptualization, J.-Y.Z.; D.-N.Y.; X.-D.X.; J.-Y.G.; methodology, J.-Y.Z.; D.-N.Y.; X.-D.X.; Y.-Y.C.; statistical analysis, J.-Y.Z.; J.-Y.G.; Z.-Y.Z.; Y.-Y.C.; investigation, D.-N.Y.; Y.-R.C.; data curation, J.-Y.G.; X.-Y.D.; Y.-Y.C.; K.B.S.; Writing—Original draft preparation, Z.-Y.Z.; J.-Y.G.; K.B.S.; Writing—Review and editing, J.-Y.Z.; D.-N.Y.; X.-D.X.; J.-Y.G.; Y.-Y.C.; K.B.S.; maps and graphics, Y.-R.C.; project administration, J.-Y.Z.; D.-N.Y.; K.B.S.; funding acquisition, J.-Y.Z.; D.-N.Y. All authors have read and agreed to the published version of the manuscript.

Reference

1. Barber-James, H.M.; Gattolliat, J.-L.; Sartori, M.; Hubbard, M.D. Global diversity of mayflies (Ephemeroptera, Insecta) in freshwater. *Hydrobiologia* **2008**, *595*, 339-350.
2. Bauernfeind, E.; Soldan, T. *The mayflies of Europe (Ephemeroptera)*; Apollo Books: Ollerup, Denmark, 2012.
3. Jacobus, L.M.; Macadam, C.R.; Sartori, M. Mayflies (Ephemeroptera) and Their Contributions to Ecosystem Services. *Insects* **2019**, *10*, 170.
4. Kukalová-Peck, J. Fossil history and the evolution of hexapod structures. In *The Insects of Australia. A Textbook for Students and Research Workers*, Naumann, I.D., Ed.; Melbourne University: Melbourne, Australia, 1991; Volume 1, pp. 141-179.

5. Thomas, J.A.; Trueman, J.W.H.; Rambaut, A.; Welch, J.J. Relaxed phylogenetics and the Palaeoptera problem: resolving deep ancestral splits in the insect phylogeny. *Syst. Biol.* **2013**, *62*, 285-297.
6. Kukalová-Peck, J. Fossil history and the evolution of Hexapod structures. In *The Insects of Australia: A Textbook for Students and Research Workers*, Naumann, I.D.C., P B; Lawrence, J F; Nielsen, E S; Spradberry, J P; Taylor, R W; Whitten, M J; Littlejohn, M J;, Ed.; CSIRO and Melbourne Univ: Melbourne, 1991; pp. 141-179.
7. Webb, J.M.; McCafferty, W.P. Heptageniidae of the world. Part II: key to the genera. *Can. J. Arthropod. Identif.* **2008**, *7*, 1-55.
8. McCafferty, W.P. Toward a phylogenetic classification of the Ephemeroptera (Insecta): a commentary on systematics. *Ann. Entomol. Soc. Am.* **1991**, *84*, 343-360.
9. McCafferty, W.P. Ephemeroptera. In *Nomina Insecta Nearctica, a checklist of the insects of North America*, Poole, R.W., Gentili, P., Eds.; Entomological Information Services: Maryland, 1997; Volume 4, pp. 89-117.
10. Kluge, N. *The Phylogenetic System of Ephemeroptera*; Kluwer Academic: Dordrecht, The Netherlands, 2004.
11. Kluge, N.J. Phylogeny and higher classification of Ephemeroptera. *Zoosystematica Rossica* **1998**, *72*, 255-269.
12. Ogden, T.H.; Gattolliat, J.L.; Sartori, M.; Staniczek, A.H.; Soldán, T.; Whiting, M.F. Towards a new paradigm in mayfly phylogeny (Ephemeroptera): combined analysis of morphological and molecular data. *Syst. Entomol.* **2009**, *34*, 616-634.
13. Wang, T.Q.; McCafferty, W.P. Heptageniidae (Ephemeroptera) of the world. Part I: phylogenetic higher classification. *Trans. Am. Entomol. Soc.* **2004**, *130*, 11-45.
14. Traver, J.R. Part II, Systematic. In *The Biology of Mayflies*, Needham, J., Traver, J., Hsu, Y., Eds.; Comstock Publishing Co.: New York, 1935; pp. 239-739.
15. Ogden, T.H.; Breinholt, J.W.; Bybee, S.M.; Miller, D.B.; Sartori, M.; Shiozawa, D.; Whiting, M.F. Mayfly Phylogenomics: Initial Evaluation of Anchored Hybrid Enrichment Data for the order Ephemeroptera. *Zoosymposia* **2019**, *16*, 167-181.
16. Zembrzuski, D.C.; Anderson, F.E. Clarifying the phylogenetic relationships and taxonomy of *Stenonema*, *Stenacron* and *Maccaffertium*, three common eastern North American mayfly genera. *Mol. Phylogenet. Evol.* **2018**, *128*, 212-220.
17. Boore, J.L. Animal mitochondrial genomes. *Nucleic Acids Res.* **1999**, *27*, 1767-1780.
18. Cameron, S.L. Insect mitochondrial genomics: Implications for evolution and phylogeny. *Annu. Rev. Entomol.* **2014**, *59*, 95-117.
19. Zhang, L.P.; Cai, Y.Y.; Yu, D.N.; Storey, K.B.; Zhang, J.Y. Gene characteristics of the complete mitochondrial genomes of *Paratoxodera polyacantha* and *Toxodera hauseri* (Mantodea: Toxoderidae). *PeerJ* **2018**, *6*, e4595.
20. Boore, J.L. The use of genome-level characters for phylogenetic reconstruction. *Trends Ecol. Evol.* **2006**, *21*, 439-446.
21. Cheng, X.F.; Zhang, L.P.; Yu, D.N.; Storey, K.B.; Zhang, J.Y. The complete mitochondrial genomes of four cockroaches (Insecta: Blattodea) and phylogenetic analyses within cockroaches. *Gene* **2016**, *586*, 115-122.
22. Ma, Y.; He, K.; Yu, P.; Yu, D.; Cheng, X.; Zhang, J. The complete mitochondrial genomes of three bristletails (Insecta: Archaeognatha): the paraphyly of Machilidae and insights into archaeognathan phylogeny. *PLoS ONE* **2015**, *10*, e0117669.
23. Wang, J.; Dai, X.Y.; Xu, X.D.; Zhang, Z.Y.; Yu, D.N.; Storey, K.B.; Zhang, J.Y. The complete mitochondrial genomes of five longicorn beetles (Coleoptera: Cerambycidae) and phylogenetic relationships within Cerambycidae. *PeerJ* **2019**, *7*, e7633.
24. Cameron, S.L.; Dowton, M.; Castro, L.R.; Ruberu, K.; Whiting, M.F.; Austin, A.D.; Diement, K.; Stevens, J. Mitochondrial genome organization and phylogeny of two vespid wasps. *Genome* **2008**, *51*, 800-808.
25. Jiang, P.; Li, H.; Song, F.; Cai, Y.; Wang, J.Y.; Liu, J.P.; Cai, W.Z. Duplication and remolding of tRNA genes in the mitochondrial genome of *Reduvius tenebrosus* (Hemiptera: Reduviidae). *Int. J. Mol. Sci.* **2016**, *17*, 951.
26. Zhang, L.P.; Cai, Y.Y.; Yu, D.N.; Storey, K.B.; Zhang, J.Y. The complete mitochondrial genome of *Psychomantis borneensis* (Mantodea: Hymenopodidae). *Mitochondrial DNA B* **2018**, *3*, 42-43.

27. Zhang, L.P.; Ma, Y.; Yu, D.N.; Storey, K.B.; Zhang, J.Y. The mitochondrial genomes of *Statilia maculata* and *S. nemoralis* (Mantidae: Mantinae) with different duplications of *trnR* genes. *Int. J. Biol. Macromol.* **2019**, *121*, 839-845.
28. Zhang, L.P.; Yu, D.N.; Storey, K.B.; Cheng, H.Y.; Zhang, J.Y. Data for praying mantis mitochondrial genomes and phylogenetic constructions within Mantodea. *Data in Brief* **2018**, *21*, 1277-1285.
29. Xin, Z.Z.; Liu, Y.; Zhang, D.Z.; Wang, Z.F.; Tang, B.P.; Zhang, H.B.; Zhou, C.L.; Chai, X.Y.; Liu, Q.N. Comparative mitochondrial genome analysis of *Spilarctia subcarnea* and other noctuid insects. *Int. J. Biol. Macromol.* **2018**, *107*, 121-128.
30. Dickey, A.M.; Kumar, V.; Morgan, J.K.; Jara-Cavieres, A.; Shatters Jr., R.G.; McKenzie, C.L.; Osborne, L.S. A novel mitochondrial genome architecture in thrips (Insecta: Thysanoptera): extreme size asymmetry among chromosomes and possible recent control region duplication. *BMC Genomics* **2015**, *16*, 439.
31. Zhang, L.P.; Yu, D.N.; Storey, K.B.; Cheng, H.Y.; Zhang, J.Y. Higher tRNA gene duplication in mitogenomes of praying mantises (Dictyoptera, Mantodea) and the phylogeny within Mantodea. *Int. J. Biol. Macromol.* **2018**, *111*, 787-795.
32. Xu, X.D.; Jia, Y.Y.; Cao, S.S.; Zhang, Z.Y.; Storey, K.B.; Yu, D.N.; Zhang, J.Y. Six complete mitochondrial genomes of mayflies from three genera of Ephemerellidae (Insecta: Ephemeroptera) with inversion and translocation of *trnI* rearrangement and their phylogenetic relationships. *PeerJ* **2020**, *8*, e9740.
33. Zhang, J.Y.; Zhou, C.F.; Gai, Y.H.; Song, D.X.; Zhou, K.Y. The complete mitochondrial genome of *Parafronurus youi* (Insecta: Ephemeroptera) and phylogenetic position of the Ephemeroptera. *Gene* **2008**, *424*, 18-24.
34. Gao, X.Y.; Zhang, S.S.; Zhang, L.P.; Yu, D.N.; Zhang, J.Y.; Cheng, H.Y. The complete mitochondrial genome of *Epeorus herklotsi* (Ephemeroptera: Heptageniidae) and its phylogeny. *Mitochondrial DNA B* **2018**, *3*, 303-304.
35. Tang, M.; Tan, M.H.; Meng, G.L.; Yang, S.Z.; Su, X.; Liu, S.L.; Song, W.H.; Li, Y.Y.; Wu, Q.; Zhang, A.B. Multiplex sequencing of pooled mitochondrial genomes-a crucial step toward biodiversity analysis using mito-metagenomics. *Nucleic Acids Res.* **2014**, *42*, e166.
36. Li, D.; Qin, J.C.; Zhou, C.F. The phylogeny of Ephemeroptera in Pterygota revealed by the mitochondrial genome of *Siphluriscus chinensis* (Hexapoda: Insecta). *Gene* **2014**, *545*, 132-140.
37. Cai, Y.Y.; Gao, Y.J.; Zhang, L.P.; Yu, D.N.; Storey, K.B.; Zhang, J.Y. The mitochondrial genome of *Caenis* sp. (Ephemeroptera: Caenidae) and the phylogeny of Ephemeroptera in Pterygota. *Mitochondrial DNA B* **2018**, *3*, 577-579.
38. Ye, Q.M.; Zhang, S.S.; Cai, Y.Y.; Storey, K.B.; Yu, D.N.; Zhang, J.Y. The complete mitochondrial genome of *Isonychia kiangsinensis* (Ephemeroptera: Isonychiidae). *Mitochondrial DNA B* **2018**, *3*, 541-542.
39. Song, N.; Li, X.X.; Yin, X.M.; Li, X.H.; Yin, J.; Pan, P.L. The mitochondrial genomes of palaeopteran insects and insights into the early insect relationships. *Sci. Rep.* **2019**, *9*, 17765.
40. Cao, S.S.; Xu, X.D.; Jia, Y.Y.; Guan, J.Y.; Storey, K.B.; Yu, D.N.; Zhang, J.Y. The complete mitochondrial genome of *Choroterpides apiculata* (Ephemeroptera: Leptophlebiidae) and its phylogenetic relationships. *Mitochondrial DNA B* **2020**, *5*, 1159-1160.
41. Xu, X.D.; Jia, Y.Y.; Dai, X.Y.; Ma, J.L.; Storey, K.B.; Zhang, J.Y.; Yu, D.N. The mitochondrial genome of *Caenis* sp. (Ephemeroptera: Caenidae) from Fujian and the phylogeny of Caenidae within Ephemeroptera. *Mitochondrial DNA B* **2020**, *5*, 192-193.
42. Ballard, J.W.O.; Kreitman, M. Is mitochondrial DNA a strictly neutral marker? *Trends Ecol. Evol.* **1995**, *10*, 485-488.
43. Cai, Y.T.; Li, Q.; Zhang, J.Y.; Storey, K.B.; Yu, D.N. Characterization of the mitochondrial genomes of two toads, *Anaxyrus americanus* (Anura: Bufonidae) and *Bufotes pewzowi* (Anura: Bufonidae), with phylogenetic and selection pressure analyses. *PeerJ* **2020**, *8*, e8901.
44. Yang, Y.X.; Xu, S.X.; Xu, J.X.; Guo, Y.; Yang, G. Adaptive evolution of mitochondrial energy metabolism genes associated with increased energy demand in flying insects. *PLoS ONE* **2014**, *9*, e99120.

45. Li, X.D.; Jiang, G.F.; Yan, L.Y.; Li, R.; Mu, Y.; Deng, W.A. Positive selection drove the adaptation of mitochondrial genes to the demands of flight and high-altitude environments in grasshoppers. *Front. Genet.* **2018**, *9*, 605.
46. Bottová, K.; Derka, T.; Svitok, M. Population dynamics of mayflies in a constant temperature spring stream in West Carpathians. *Limnologica* **2013**, *43*, 469-474.
47. Scherr, M.A.; Wooster, D.E.; Rao, S. Effects of temperature on growth rate and behavior of *Epeorus albertae* (Ephemeroptera: Heptageniidae) nymphs. *Environ. Entomol.* **2010**, *39*, 2017-2024.
48. Zhai, Y.Y.; Huang, G.; Wang, X.Q.; Zhou, X.; Lu, C.; Li, Z. Future projections of temperature changes in Ottawa, Canada through stepwise clustered downscaling of multiple GCMs under RCPs. *Climate Dynamics* **2019**, *52*, 3455-3470.
49. Gao, X.Y.; Cai, Y.Y.; Yu, D.N.; Storey, K.B.; Zhang, J.Y. Characteristics of the complete mitochondrial genome of *Suhpalacsa longialata* (Neuroptera, Ascalaphidae) and its phylogenetic implications. *PeerJ* **2018**, *6*, e5914.
50. Simon, C.; Buckley, T.R.; Frati, F.; Stewart, J.B.; Beckenbach, A.T. Incorporating molecular evolution into phylogenetic analysis, and a new compilation of conserved polymerase chain reaction primers for animal mitochondrial DNA. *Annu. Rev. Ecol. Evol. Syst.* **2006**, *37*, 545-579.
51. Simon, C.; Frati, F.; Beckenbach, A.; Crespi, B.; Liu, H.; Flook, P. Evolution, weighting, and phylogenetic utility of mitochondrial gene sequences and a compilation of conserved polymerase chain reaction primers. *Ann. Entomol. Soc. Am.* **1994**, *87*, 651-701.
52. Lalitha, S. Primer premier 5. *Biotech. Softw. Internet Rep.* **2000**, *1*, 270-272.
53. Burland, T.G. DNASTAR's Lasergene sequence analysis software. In *Bioinformatics Methods and Protocols*, Misener, S., Krawetz, S.A., Eds.; Humana Press: Totowa, NJ, 1999; pp. 71-91.
54. Bernt, M.; Donath, A.; Jühling, F.; Externbrink, F.; Florentz, C.; Fritzsch, G.; Pütz, J.; Middendorf, M.; Stadler, P.F. MITOS: Improved de novo metazoan mitochondrial genome annotation. *Mol. Phylogenet. Evol.* **2013**, *69*, 313-319.
55. Thompson, J.D.; Gibson, T.J.; Plewniak, F.; Jeanmougin, F.; Higgins, D.G. The CLUSTAL_X windows interface: Flexible strategies for multiple sequence alignment aided by quality analysis tools. *Nucleic Acids Res.* **1997**, *25*, 4876-4882.
56. Kumar, S.; Stecher, G.; Tamura, K. MEGA7: molecular evolutionary genetics analysis version 7.0 for bigger datasets. *Mol. Biol. Evol.* **2016**, *33*, 1870-1874.
57. Perna, N.T.; Kocher, T.D. Patterns of nucleotide composition at fourfold degenerate sites of animal mitochondrial genomes. *J. Mol. Evol.* **1995**, *41*, 353-358.
58. Benson, G. Tandem repeats finder: a program to analyze DNA sequences. *Nucleic Acids Res.* **1999**, *27*, 573-580.
59. Reuter, J.S.; Mathews, D.H. RNAstructure: software for RNA secondary structure prediction and analysis. *BMC Bioinf.* **2010**, *11*, 129.
60. Katoh, K.; Standley, D.M. MAFFT multiple sequence alignment software version 7: improvements in performance and usability. *Mol. Biol. Evol.* **2013**, *30*, 772-780.
61. Castresana, J. Selection of conserved blocks from multiple alignments for their use in phylogenetic analysis. *Molecular Biology and Evolution* **2000**, *17*, 540-552.
62. Lanfear, R.; Calcott, B.; Ho, S.Y.; Guindon, S. Partitionfinder: combined selection of partitioning schemes and substitution models for phylogenetic analyses. *Mol. Biol. Evol.* **2012**, *29*, 1695-1701.
63. Stamatakis, A. RAxML version 8: a tool for phylogenetic analysis and post-analysis of large phylogenies. *Bioinformatics* **2014**, *30*, 1312-1313.
64. Ronquist, F.; Teslenko, M.; Mark, P.V.D.; Ayres, D.L.; Darling, A.; Höhna, S.; Larget, B.; Liu, L.; Suchard, M.A.; Huelsenbeck, J.P. MrBayes 3.2: efficient Bayesian phylogenetic inference and model choice across a large model space. *Syst. Biol.* **2012**, *61*, 539-542.
65. Gao, F.L.; Chen, C.J.; Arab, D.A.; Du, Z.G.; He, Y.H.; Ho, S.Y.W. EasyCodeML: A visual tool for analysis of selection

using CodeML. *Ecol. Evol.* **2019**, *9*, 3891-3898.
66. Yang, Z. Likelihood ratio tests for detecting positive selection and application to primate lysozyme evolution. *Mol Biol Evol* **1998**, *15*, 568-573.
67. Zhang, J.Z.; Nielsen, R.; Yang, Z.H. Evaluation of an improved branch-site likelihood method for detecting positive selection at the molecular level. *Mol. Biol. Evol.* **2005**, *22*, 2472-2479.
68. The UniProt Consortium. UniProt: the universal protein knowledgebase. *Nucleic Acids Res.* **2018**, *46*, 2699.
69. Waterhouse, A.; Bertoni, M.; Bienert, S.; Studer, G.; Tauriello, G.; Gumienny, R.; Heer, F.T.; de Beer, T.A P.; Rempfer, C.; Bordoli, L.; et al. SWISS-MODEL: homology modelling of protein structures and complexes. *Nucleic Acids Res.* **2018**, *46*, W296-W303.
70. McDunnough, J. Notes on North American Ephemeroptera with descriptions of a new species. *Can. Entomol.* **1926**, *58*, 184-196.
71. Wu, T.; You, D.S. A new species of the genus *Cinygmina* from China(Ephemeroptera: Ecdyoneuridae). *Acta Zootaxonomica Sinica* **1986**, *11*, 280-282.
72. Zhou, C.F.; Zheng, L.Y. The genus *Cinygmina*(Ephemeroptera:Heptageniidae) in China, with a description of a new species. *Acta Entomologica Sinica* **2003**, *46*, 755-760.
73. You, D.; Wu, T.; Gui, H.; Hsu, Y. Two new species and diagnostic characters of genus *Cinygmina* (Ephemeroptera: Ecdyonuridae). *Journal of Nanjing Normal University (Natural Science)* **1981**, *3*, 26-30.
74. Walker, F. Part III. - (Termitidae-Ephemeridae). In *Catalogue of the specimens of neuropterous insects in the collection of the British Museum*; British Museum: London, 1853; pp. 477-585.
75. Say, T. Descriptions of new North American neuropterous insects and observation on some already described. *J. Acad Nat. Sci. Phila.* **1839**, *8*, 9-46.
76. Say, T. Descriptions of insects belonging to the order Neuroptera Linne and Latreille, collected by the expedition under the command of Major Long. *Western Quarterly Reporter* **1823**, *2*, 160-165.
77. Gui, H.; Zhang, J. A new species of genus Epeorus Eaton (Ephemeroptera: Heptageniidae). *Acta zootaxon. sin.* **1992**, *17*, 61-63.
78. Tomita, K.; Ueda, T.; Ishiwa, S.; Crain, P.F.; McCloskey, J.A.; Watanabe, K. Codon reading patterns in *Drosophila melanogaster* mitochondria based on their tRNA sequences: a unique wobble rule in animal mitochondria. *Nucleic Acids Res.* **1999**, *27*, 4291-4297.
79. Watanabe, K.; Yokobori, S.-I. tRNA modification and genetic code variations in animal mitochondria. *J. Nucleic Acids* **2011**, *2011*, 1-12.
80. Cameron, S.L.; Whiting, M.F. The complete mitochondrial genome of the tobacco hornworm, *Manduca sexta*,(Insecta: Lepidoptera: Sphingidae), and an examination of mitochondrial gene variability within butterflies and moths. *Gene* **2008**, *408*, 112-123.
81. Li, H.; Liu, H.; Shi, A.; Stys, P.; Zhou, X.; Cai, W. The complete mitochondrial genome and novel gene arrangement of the unique-headed bug *Stenopirates* sp. (Hemiptera: Enicocephalidae). *PLoS ONE* **2012**, *7*, e29419.
82. Arndt, A.; Smith, M.J. Mitochondrial gene rearrangement in the sea cucumber genus *Cucumaria*. *Mol. Biol. Evol.* **1998**, *15*, 1009-1016.
83. Excoffier, L. Evolution of human mitochondrial DNA: evidence for departure from a pure neutral model of populations at equilibrium. *J. Mol. Evol.* **1990**, *30*, 125-139.
84. Tan, M.H.; Zhang, R.; Hardman, C.; Zhou, X. Mitochondrial genome of *Hylaeus dilatatus* (Hymenoptera: Colletidae). *Mitochondrial DNA A* **2016**, *27*, 3975-3976.
85. Zhang, B.; Ma, C.; Edwards, O.; Fuller, S.; Kang, L. The mitochondrial genome of the Russian wheat aphid *Diuraphis*

noxia: large repetitive sequences between *trnE* and *trnF* in aphids. *Gene* **2014**, *533*, 253-260.
86. Crozier, R.H.; Crozier, Y.C. The mitochondrial genome of the honeybee *Apis mellifera*: Complete sequence and genome organization. *Genetics* **1993**, *133*, 97-117.
87. Dotson, E.M.; Beard, C.B. Sequence and organization of the mitochondrial genome of the Chagas disease vector, *Triatoma dimidiata*. *Insect Mol. Biol.* **2001**, *10*, 205-215.
88. Lunt, D.H.; Hyman, B.C. Animal mitochondrial DNA recombination. *Nature* **1997**, *387*, 247.
89. Zhang, H.L.; Ye, F. Comparative mitogenomic analyses of praying mantises (Dictyoptera, Mantodea): origin and evolution of unusual intergenic gaps. *Int. J. Biol. Sci.* **2017**, *13*, 367-382.
90. Roberti, M.; Polosa, P.L.; Bruni, F.; Musicco, C.; Gadaleta, M.N.; Cantatore, P. DmTTF, a novel mitochondrial transcription termination factor that recognises two sequences of *Drosophila melanogaster* mitochondrial DNA. *Nucleic Acids Res.* **2003**, *31*, 1597-1604.
91. Zheng, B.Y.; Cao, L.J.; Tang, P.; van Achterberg, K.; Hoffmann, A.A.; Chen, H.Y.; Chen, X.X.; Wei, S.J. Gene arrangement and sequence of mitochondrial genomes yield insights into the phylogeny and evolution of bees and sphecid wasps (Hymenoptera: Apoidea). *Mol. Phylogenet. Evol.* **2018**, *124*, 1-9.
92. Tyagi, K.; Chakraborty, R.; Cameron, S.L.; Sweet, A.D.; Chandra, K.; Kumar, V. Rearrangement and evolution of mitochondrial genomes in Thysanoptera (Insecta). *Sci. Rep.* **2020**, *10*, 695.
93. Scheffler, I.E. Molecular genetics of succinate:quinone oxidoreductase in eukaryotes. *Prog. Nucleic Acid Res. Mol. Biol.* **1998**, *60*, 267-315.
94. Shen, Y.J.; Kou, Q.; Zhong, Z.X.; Li, X.Z.; He, L.S.; He, S.P.; Gan, X.N. The first complete mitogenome of the South China deep-sea giant isopod *Bathynomus* sp. (Crustacea: Isopoda: Cirolanidae) allows insights into the early mitogenomic evolution of isopods. *Ecol. Evol.* **2017**, *7*, 1869-1881.
95. Brandt, U. Energy converting NADH:quinone oxidoreductase (complex I). *Annu. Rev. Biochem.* **2006**, *75*, 69-92.
96. Kühlbrandt, W. Structure and function of mitochondrial membrane protein complexes. *BMC Biol.* **2015**, *13*, 89.
97. Wirth, C.; Brandt, U.; Hunte, C.; Zickermann, V. Structure and function of mitochondrial complex I. *Biochim. Biophys. Acta* **2016**, *1857*, 902-914.
98. Bridges, H.R.; Birrell, J.A.; Hirst, J. The mitochondrial-encoded subunits of respiratory complex I (NADH:ubiquinone oxidoreductase): identifying residues important in mechanism and disease. *Biochem. Soc. Trans.* **2011**, *39*, 799-806.
99. Mathiesen, C.; Hägerhäll, C. Transmembrane topology of the NuoL, M and N subunits of NADH:quinone oxidoreductase and their homologues among membrane-bound hydrogenases and bona fide antiporters. *Biochim. Biophys. Acta, Bioenerg.* **2002**, *1556*, 121-132.
100. Andreu, A.L.; Hanna, M.G.; Reichmann, H.; Bruno, C.; Penn, A.S.; Tanji, K.; Pallotti, F.; Iwata, S.; Bonilla, E.; Lach, B.; et al. Exercise intolerance due to mutations in the cytochrome *b* gene of mitochondrial DNA. *N. Engl. J. Med.* **1999**, *341*, 1037-1044.
101. Bourens, M.; Barrientos, A. A *CMC1*-knockout reveals translation-independent control of human mitochondrial complex IV biogenesis. *EMBO Rep.* **2017**, *18*, 477-494.
102. DePristo, M.A.; Weinreich, D.M.; Hartl, D.L. Missense meanderings in sequence space: a biophysical view of protein evolution. *Nat. Rev. Genet.* **2005**, *6*, 678-687.
103. Bromberg, Y.; Rost, B. Correlating protein function and stability through the analysis of single amino acid substitutions. *BMC Bioinf.* **2009**, *10*, S8.
104. Zhou, D., Wang, Y. Y., Sun, J. Z., Han, Y. K., & Zhou, C. F. The complete mitochondrial genome of *Paegniodes cupulatus* (Ephemeroptera: Heptageniidae). *Mitochondrial DNA Part A*. **2016**, *27*(2), 925-926.
