# Supplementary material for: Insight into the Phylogenetic Relationships among Three Subfamilies within Heptageniidae (Insecta: Ephemeroptera) along with Low-Temperature Selection Pressure Analyses Using Mitogenomes
Source: Insects. 2021 Jul 19;12(7):656. doi: 10.3390/insects12070656 (PMC8307263; doi:10.3390/insects12070656)
Supplement: Supplementary file 1 [file insects-12-00656-s001.zip › Table S1. Specific primers used to amplify the Ephemeroptera mitochondrial genomes.pdf]

Table S1. Specific primers used to amplify the Heptageniidae mitochondrial genomes.

Species	Primer Name	Sequence (5'-3')	Product length (nt)
M. modestum	03FY62-J-2318	TATTCTGATTCTTCGGACA	2400
	03FY62-N-4600	AGAAGAGTTAGTGACAGGCT	
	03FY62-J-5600	TCTCTTCAAAACAGGTAATG	3500
	03FY62-N-9000	TACTGGTGGCTATTTTCTAT	
	03FY62-J-9300	ATTCTGGGTTCTAAAAGTGC	2300
	03FY62-N-11600	TAAAGGAAGTAAGTGACGGT	
	03FY62-J-14650	CCAGATTTCATAACTTCGC	1000
	03FY62-N-286	CTTCCTCCATCCACTACAT	
M. vicarium	03FY39-J-3228	CTTGATGAAGTAAGCGACC	1500
	03FY39-N-5226	GTGTTCAAAGTAATGCCGA	
	03FY39-J-5184	CTATCGCAGATGCTGTGTA	3300
	03FY39-N-8502	TAGGGTTAGTGTTAGGGGG	
	03FY39-J-8898	TCACTAAACATCCTCCAACT	2500
	03FY39-N-11424	CGTAGGCGAATAGGAAATA	
	03FY39-J-14579	CCAGATTTCATAACTTCGC	1000
	03FY39-N-384	GGCTGAGATGGTGATTAGG	
M. mediopunctatum	03FY33-J-3196	TACACACCGCTACCTTCTTG	1500
	03FY33-N-4989	CAGGAGACAAACTGCTATGAA	
	03FY33-J-5010	CCTATTAGTCTGCCTCCTTC	3500
	03FY33-N-8834	TTAGTTCACCTTTGATTACCC	
	03FY33-J-9012	TAAAGGTAAAGATGCCAGTA	2000
	03FY33-N-11059	AAAAGTGAAGTAAGGGTGG	
	03FY33-J-14217	GCACCTTGACCTGACATCTA	1000
	03FY33-N-314	AGGGGAATGAAGGATAAGAG	
S. interpunctatum	03FY34-J-3164	GAGCAACTCACCTTTTTCC	1700
	03FY34-N-4920	TCGGGTAATGTCTCGTCAC	
	03FY34-J-5499	TTTGTAGATGTGGTATGGCTC	2000
	03FY34-N-7418	TACTTTAGTTACGGCTGGG	
	03FY34-J-7351	AACATAGTAAGTCCCGCAAT	2000
	03FY34-N-9307	TGAGATTTTGGATTTGTGGT	
	03FY34-J-9325	AACCACAAATCCAAAATCTC	1700
	03FY34-N-11087	TCTACATCACTGTTTACCCCT	
	03FY34-J-14094	ACCTTCCTTTCAACTTTCACC	1000
	03FY34-N-277	AAGTAACCGAGTGGGATGG	
S. femoratum	03FY36-J-3191	CCTACACCCACCGATACCT	1400
	03FY36-N-4628	TGCGAAAACATAGGATTGG	
	03FY36-J-5237	TTATGAATACATTGAAGCCC	2000
	03FY36-N-7444	ATAACAAAGAGAGCCCAGAT	
	03FY36-J-7466	GGGCTCTCTTTGTTATTGC	1500
	03FY36-N-9000	ACACTTTGCTTGCTTCGTT	
	03FY36-J-9279	CTCAACAGAATCAACCCAA	2000
	03FY36-N-11225	GAAGAATAGCGTAAGCGAA	

	03FY36-J-14867	CGATTAGAACTCTCAAAAAAG	1000
	03FY36-N-344	GAGGATGTCTCAAAGATGGG	
L. aphrodite	03FY51-J-2386	CATCATTAGCCAGGAAAGAG	2500
	03FY51-N-5058	CTTGGTAGGTTCCTTCTCG	
	03FY51-J-5500	ATTTTGAACACTTCTCCGC	3000
	03FY51-N-8886	TAGATTGGTTGGTGGGTGT	
	03FY51-J-9006	CAACAAAACTCCTGCTAAA	2000
	03FY51-N-11091	ATCCTCCTCAAACTCACTG	
	03FY51-J-14314	TAATGTAACCCATCTCCCC	1000
	03FY51-N-402	AAATAGCCACAAAAGTCCC	
A. yixingensis	06BF03-J-3429	CCTCCTAGATGAAGTTAGAGAC	3000
	06BF03-N-7534	TATATGTGCTGGTGCTGTTA	
	06BF03-J-7124	ATAACAGCACCAGCACATAT	1800
	06BF03-N-8962	ACTCTATTGGCTTCGTTACC	
	06BF03-J-9276	GGTAACGAAGCCAATAGAGT	2000
	06BF03-N-11134	TCAGGTTGAATATGGACAGG	
	06BF03-J-13571	AATCCACCTTCCAACTACTT	1000
	06BF03-N-938	CCAACTTAATCCTTCCATCAC	
A. furcata	08BF03-J-2814	ATGTTGTGTCTTCAGTTGGA	3000
	08BF03-N-5963	AGGATTAAGGCAGGTTGATG	
	08BF03-J-6001	TCATCAACCTGCCTTAATCC	2500
	08BF03-N-8865	GAGTTCTGCTGAAGTTAGGA	
	08BF03-J-9521	ACTTAACGACTGATTGGCTA	1600
	08BF03-N-11052	AGGAAGAGGAGGTGAACTAT	
	08BF03-J-14578	CTCTTACTACTCAGGCTCAC	1000
	08BF03-N-732	CCAAGTGGTTGATTGATGAA	
A. rubromaculata	08BF02-J-3309	CCTGAGCAGTCCCATCTTT	1100
	08BF02-N-4492	GTCACGGGCTTTGGTCTAC	
	08BF02-J-5161	TCTGCTGGTCATCACTTTG	3000
	08BF02-N-8533	GGTTACGGACTTTTGCGAG	
	08BF02-J-8752	CCGTAACCTCCTAACTTCAG	2400
	08BF02-N-11543	AGAGAACCAATGCGATAACT	
	08BF02-J-14357	CCACCTTCCAATCATTATTCC	1500
	08BF02-N-391	CTTAATCCTTCCATAACTCCAG	
	08BF02-J-15417	ATGGAAAACTACGGGTCAC	1000
	08BF02-N-594	GTGGGACCATCCTACTTGA	
Afronurus sp. YW01BF06	01BF06-J-3255	TTGCCTTACCTTCATTACGA	3000
	01BF06-N-7464	CGGAGGATTAGTAGTCTTGG	
	01BF06-J-7592	AGCAGCCAAGACTACTAATC	1400
	01BF06-N-8942	GCCTGTTAGAGGTTCTATGAT	
	01BF06-J-9458	CGACTGACTAGCCATAATCA	1600
	01BF06-N-11158	TAGAACGGAGAATAGCATAGG	
	01BF06-J-14317	ACTCTTCTAACGGCGGTATA	1500
	01BF06-N-822	ATAAGGCTGCTAACATCCAA	

	01BF06-J-14178	GCCTTATTGTAATCCATCTCC	1200
	01BF06-N-724	AGGACTACGGACATTCTTAA	
Afronurus sp. LS53BF04	53BF04-J-3254	CACTTCCTTCCCTTCGTTTAC	3000
	53BF04-N-7393	GGTGTTTGGTTGTGTTAGCAG	
	53BF04-J-7322	AGGAGTGGACCAAAGCAGAA	1300
	53BF04-N-8675	GAGGTTATGGACTTTTGCGA	
	53BF04-J-9128	TATGAACCAGGTGTCAACTC	2000
	53BF04-N-11056	AGGAAGAGGAGGTGAACTAT	
	53BF04-J-14787	GACATCTCGGAACTCTTGAA	1200
	53BF04-N-158	GAGCAGGATTCTAGGATCATT	
	53BF04-J-14275	AATCCACCTTCCAACCATTA	1500
	53BF04-N-650	TAGTGTACGAAGCACAAGAG	
E. dayongensis	18BF01-J-3105	AAGCAAGTAGTGGTCTCTTA	1100
	18BF01-N-4287	GTAACAATGTAGGTGTGGAG	
	18BF01-J-3352	GTTAGACGAAGTTAGAGATCCT	1600
	18BF01-N-4955	AACAATGTAGGTGTGGAGAC	
	18BF01-J-5270	CCATCCTACAAGCCTATGAA	2000
	18BF01-N-7368	ATTGAGGGAACTGGAGTAAC	
	18BF01-J-7544	ACAGCCTACTAATCCTATTTCC	1500
	18BF01-N-9105	GTATCAACCTGAACGAGTCC	
	18BF01-J-9279	GTACTGATTGGCTTGCTATA	1900
	18BF01-N-11255	ATTCTGGTTGGATATGAACG	
	18BF01-J-14264	ACCTTGACCTGACATCTTTC	1000
	18BF01-N-486	GGCTTAGAGTTACTACACCAA	
	18BF01-J-14101	ACGACCTCATTCCAGTGTAA	1000
	18BF01-N-423	CAATCAAGACCTTCCATAACTC	
Epeorus sp. LA03FY06	03FY06-J- 3423	TACATAATCCCCACCCA	1200
	03FY06-N-4630	AATAGCAACAGCAGACTCA	
	03FY06-J-5294	TGCTGTTTACGGCTCTAC	2000
	03FY06-N-7423	TTCCTTTTTCTGCTTGGT	
	03FY06-J-8102	TAAGAGGTCTAAACCGAAT	9000
	03FY06-N-9008	TTTTATACATTGTTAGCATC	
	03FY06-J-9007	CAATAAAGGTAAAGATGCTAA	1900
	03FY06-10908	AAAATCGGGTAAGAGTAGC	
	03FY06-13591	TAGATACCTTGAGGACCGAT	1500
	03FY06-522	TGCCAGTTTGAGGATAGAGT	
	03FY06-J-14184	GCACCTTGACCTGACATACT	1000
	03FY06-N-1059	TAAAGTAGCCGAGGCAAAC	
Heptageniidae sp. YW03BF02	03BF02-J-3546	ACCTCCTAGACGAAGTAAGA	1100
	03BF02-N-4658	AATCATTGAACGAGTCCTCT	
	03BF02-J-5547	GTAGATGTAGTGTGGTTATTCC	3000
	03BF02-N-8791	GGTATGGCTTATTGCGAGTA	
	03BF02-J-9157	AAGGTAATGAAGCCAACAAC	2000
	03BF02-N-11249	AAGAGTAAGTGGACCATAGTAG	

03BF02-J-13987	ACCATAACTCTTACTACTCAGG	1000
03BF02-N-623	ATAAGGTTGCTGCCAGTATT	
