# Supplementary material for: Insight into the Phylogenetic Relationships among Three Subfamilies within Heptageniidae (Insecta: Ephemeroptera) along with Low-Temperature Selection Pressure Analyses Using Mitogenomes
Source: Insects. 2021 Jul 19;12(7):656. doi: 10.3390/insects12070656 (PMC8307263; doi:10.3390/insects12070656)
Supplement: Supplementary file 1 [file insects-12-00656-s001.zip › Table S2. The partition schemes and best-fitting models selected.pdf]

Table S2. The partition schemes and best-fit models selected

Nucleotide sequence alignments		
Subset	Subset partitions	Best model
Partition 1	*COX2*_codon1, *COX3*_codon1, *ATP6*_codon1, *Cyt b*_codon1	GTR+I+G
Partition 2	*COX1*_codon2, *COX2*_codon2, *ATP6*_codon2, *COX3*_codon2, *Cyt b*_codon2	GTR+I+G
Partition 3	*COX3*_codon3, *ND3*_codon3, *ND6*_codon3, *ATP6*_codon3, *ATP8*_codon3	TRN+I+G
Partition 4	*ATP8*_codon1, *ND6*_codon1, *ND2*_codon1	GTR+I+G
Partition 5	*ATP8*_codon2, *ND3*_codon2, *ND2*_codon2, *ND6*_codon2	GTR+G
Partition 6	*COX1*_codon1	GTR+I+G
Partition 7	*COX1*_codon3	HKY+I+G
Partition 8	*COX2*_codon3, *Cyt b*_codon3	GTR+I+G
Partition 9	*ND4L*_codon1, *ND3*_codon1, *ND5*_codon1, *ND1*_codon1, *ND4*_codon1	GTR+I+G
Partition 10	*ND4L*_codon2, *ND1*_codon2, *ND5*_codon2, *ND4*_codon2	GTR+I+G
Partition 11	*ND4*_codon3, *ND1*_codon3, *ND5*_codon3, *ND4L*_codon3	GTR+I+G
Partition 12	*ND2*_codon3	TIM+G
