# Supplementary material for: Insight into the Phylogenetic Relationships among Three Subfamilies within Heptageniidae (Insecta: Ephemeroptera) along with Low-Temperature Selection Pressure Analyses Using Mitogenomes
Source: Insects. 2021 Jul 19;12(7):656. doi: 10.3390/insects12070656 (PMC8307263; doi:10.3390/insects12070656)
Supplement: Supplementary file 1 [file insects-12-00656-s001.zip › Table S6. Positive selection in Branch-model.pdf]

**Table S6.** Positive selection analysis of mitochondrial protein-coding genes based on the branch model.

| Tree | Model | Ln L | Estimates of parameters | Model compared | 2ΔL | LRT P-value |
|---|---|---|---|---|---|---|
| ML | Two- ratio | -123656.793918 | ω0= 0.02021 ω1= 0.00010 | Model 0 vs. Two ratio | 176.3226 | 0.0000 |
| | Model 0 | -123744.955230 | ω= 0.02123 | Model 2 | | |
